# Engineering of a newly isolated *Bacillus tequilensis* BL01 for poly-γ-glutamic acid production from citric acid

**DOI:** 10.1186/s12934-022-01994-z

**Published:** 2022-12-29

**Authors:** Dexin Wang, Xiaoping Fu, Dasen Zhou, Jiaqi Gao, Wenqin Bai

**Affiliations:** 1 CAS Key Laboratory of Systems Microbial Biotechnology, Tianjin Institute of Industrial Biotechnology, Chinese Academy of Sciences, Tianjin, 300308 China; 2 National Center of Technology Innovation for Synthetic Biology, Tianjin, 300308 China; 3 College of Biotechnology, Tianjin University of Science and Technology, Tianjin, 300457 China; 4 University of Chinese Academy of Sciences, 19A Yuquan Road, Shijingshan District, Beijing, 100049 China

**Keywords:** Poly-γ-glutamic acid, *Bacillus tequilensis* BL01, Metabolic engineering, Gene knockout

## Abstract

**Background:**

Poly γ-glutamic acid (γ-PGA) is a promising biopolymer for various applications. For glutamic acid-independent strains, the titer of γ-PGA is too low to meet the industrial demand. In this study, we isolated a novel γ-PGA-producing strain, *Bacillus tequilensis* BL01, and multiple genetic engineering strategies were implemented to improve γ-PGA production.

**Results:**

First, the one-factor-at-a-time method was used to investigate the influence of carbon and nitrogen sources and temperature on γ-PGA production. The optimal sources of carbon and nitrogen were sucrose and (NH_4_)_2_SO_4_ at 37 °C, respectively. Second, the *sucA*, *gudB*, *pgdS*, and *ggt* genes were knocked out simultaneously, which increased the titer of γ-PGA by 1.75 times. Then, the titer of γ-PGA increased to 18.0 ± 0.3 g/L by co-overexpression of the *citZ* and *pyk* genes in the mutant strain. Furthermore, the γ-PGA titer reached 25.3 ± 0.8 g/L with a productivity of 0.84 g/L/h and a yield of 1.50 g of γ-PGA/g of citric acid in fed-batch fermentation. It should be noted that this study enables the synthesis of low (1.84 × 10^5^ Da) and high (2.06 × 10^6^ Da) molecular weight of γ-PGA by BL01 and the engineering strain.

**Conclusion:**

The application of recently published strategies to successfully improve γ-PGA production for the new strain *B. tequilensis* BL01 is reported. The titer of γ-PGA increased 2.17-fold and 1.32-fold compared with that of the wild type strain in the flask and 5 L fermenter. The strain shows excellent promise as a γ-PGA producer compared with previous studies. Meanwhile, different molecular weights of γ-PGA were obtained, enhancing the scope of application in industry.

**Supplementary Information:**

The online version contains supplementary material available at 10.1186/s12934-022-01994-z.

## Background

Poly-γ-glutamic acid (γ-PGA), one of the most promising biopolymers, consists of D- and l-glutamic acid units linked by amide bonds between α-amino and γ-carboxylic acid groups [1]. γ-PGA shows great potential for application in food [2], medicine [3], cosmetics [4], wastewater treatment [5], and agriculture [6] due to its numerous properties, including water solubility, biodegradability, nontoxicity, and biocompatibility [7, 8]. Recently, γ-PGA has shown an increasing number of promising applications. γ-PGA nanocomposite hydrogels are potentially applied for injectable tissue engineering hydrogels, tissue adhesives, and hemostatic materials [9]. The SiOx electrode using γ-PGA cross-linked by epichlorohydrin as the binder achieves high reversible capacity and outstanding cycle stability [10]. In agriculture, exogenous application of γ-PGA could significantly improve plant drought resistance by improving photosynthesis, and root development, and enriching plant growth-promoting bacteria [11].

γ-PGA is synthesized primarily by various *Bacillus* species, including *B. subtilis* [12, 13], *B. siamensis* [14, 15], *B. licheniformis* [16, 17], *B. methylotrophic* [18], *B. amyloliquefaciens* [19, 20], and *B. velezensis* [21]. Depending on the substrate used, strains can be divided into two types. The first type is glutamic acid-dependent strains that require l-glutamic acid as a direct precursor [12–15]. Supplying exogenous glutamic acid significantly increases γ-PGA production, but also increases production costs. The other type is glutamic acid-independent strains, which can synthesize γ-PGA from carbon sources such as glucose de novo [16–18, 20]. Glutamic acid-independent strains have attracted widespread attention due to their low production cost. However, low γ-PGA titers and productivity limit their industrial applications. Therefore, it is vital to obtain novel strains producing γ-PGA with high titers, yields, and productivities that will be economically feasible for industrial production.

To meet the growing demand for economical γ-PGA, an increasing number of studies have focused on metabolic engineering strategies. In *B. amyloliquefaciens*, the γ-PGA titer increased from 3.14 to 5.12 g/L by double deletion of the *cwlO* gene and the *epsA-O* cluster, as well as expression of the *Vitreoscilla* hemoglobin (*VHb*) gene [19]. Improving NADPH regeneration is another method to enhance γ-PGA production [22]. Furthermore, by reducing byproduct production by gene knockout and improving precursors synthesis by overexpressing genes related to glutamate synthesis, the yield of γ-PGA was significantly increased [19, 23, 24]. Moreover, the effect on the deletion of genes that encode enzymes that degrade γ-PGA and glutamate, such as the *pgdS*, *ggt*, *gudB*, *rocG*, and *proB* genes, was also studied [24, 25]. Interestingly, deletion of these genes was strain dependent. Different strains knocked out the same gene with varying effects on γ-PGA production.

**Fig. 1:**
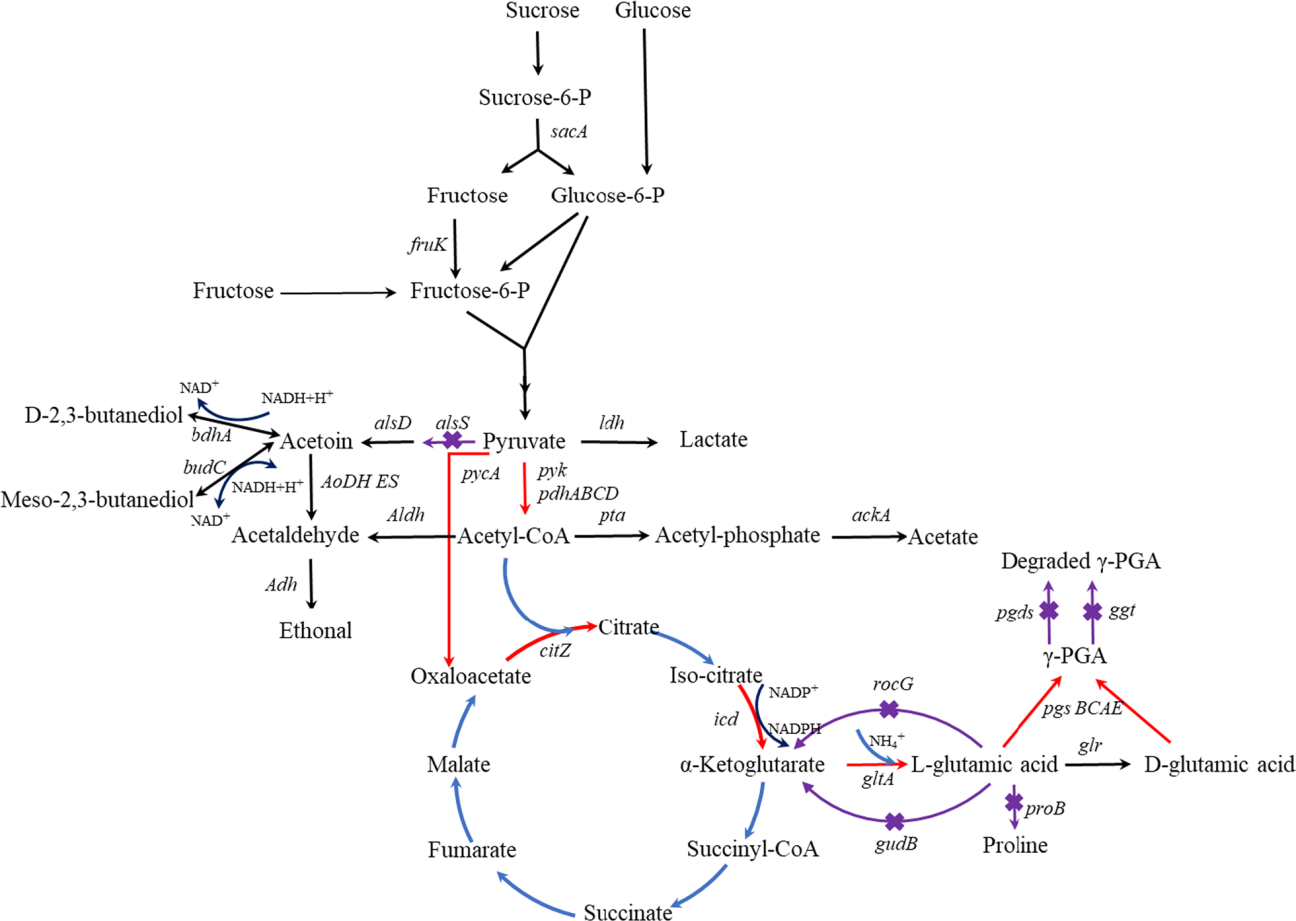
Metabolic pathways and metabolic engineering strategies to improve the production of γ-PGA in *B. tequilensis* BL01. Red arrows indicate genes that are overexpressed for γ-PGA production: pyruvate dehydrogenase (*pdhABCD*), pyruvate kinase (*pyk*), pyruvate carboxylase (*pycA*), citrate synthase II (*citZ*), isocitrate dehydrogenase (*icd*), glutamate synthase (*gltA*), poly-gamma-glutamate synthase complex (*pgsBCAE*); Purple arrows indicate genes that are deleted: acetolactate synthase (*alsS*), glutamate dehydrogenase (*rocG*), cryptic glutamate dehydrogenase (*gudB*), glutamate 5-kinase (*proB*), 2-oxoglutarate dehydrogenase E1 component (*sucA*), gamma-DL-glutamyl hydrolase (*pgdS*), membrane-bound gamma-glutamyltranspeptidase (*ggt*)

Although many studies have made some progress, most have focused on a single research bottleneck and have not studied the combination of factors affecting γ-PGA synthesis. In this study, we selected an economical, high-yield γ-PGA-producing strain, and multiple genetic engineering strategies were implemented to improve γ-PGA production. First, the components affecting γ-PGA production were optimized, and the strain was identified as a glutamic acid-independent strain. Second, the *pgdS* and *ggt* genes were deleted to reduce γ-PGA degradation. Third, the *alsS*, *sucA*, *gudB*, *proB*, and *rocG* genes were deleted to decrease the production of byproducts and improve glutamate production. Fourth, the genes involved in the synthesis of precursor and γ-PGA were overexpressed (Fig. 1). The final obtained strain of *B*. *tequilensis* BL01Δ*pgdS*Δ*ggt*Δ*sucA*Δ*gudB*:*P43*-*citZ*-*pyk* could produce γ-PGA titers of 18.0 ± 0.3 g/L in a flask and 25.2 ± 0.8 g/L in a 5 L fermenter, which were 2.12 times and 1.31 times higher than those obtained from the wild-type strain of *B*. *tequilensis* BL01, respectively.

## Materials and methods

### Strain and medium

All plasmids and strains used in this study are listed in Additional file 1: Table S1. *E. coli* DH5α cultured in LB medium containing 5 g/L NaCl, 10 g/L tryptone, and 5 g/L yeast extract with or without 20 g/L agar was used to construct the plasmid.

The *Bacillus tequilensis* BL01 strain was isolated from soybean and stored at the China General Microbiological Culture Collection Center (CGMCC23661). γ-PGA-producing strains were preliminarily screened and cultured in basal medium containing 10 g/L tryptone, 5 g/L yeast extract, 10 g/L NaCl, 20 g/L glucose, and 10 g/L monosodium glutamate. Agar (20 g/L) was added to basal medium agar plates. A strain with a high γ-PGA yield was screened, and the influencing factors were analyzed in the fermentation medium, which contained 30 g/L glucose, 20 g/L monosodium glutamate, 5 g/L (NH_4_)_2_SO_4_, 0.5 g/L K_2_HPO_4_, 0.5 g/L MgSO_4_·7H_2_O, 0.04 g/L FeCl_3_·6H_2_O, 0.104 g/L MnSO_4_·H_2_O, 0.15 g/L CaCl_2_, and 0.5 g/L NaCl. For the fermentation of genetically modified bacteria, 10 g/L sodium citrate was added to the medium instead of 20 g/L monosodium glutamate.

The working concentrations of chloramphenicol, ampicillin, kanamycin and β-d-1-thiogalactopyranoside (IPTG) in this study were 5 μg/mL, 100 μg/mL, 40 μg/mL, and 0.1 mM, respectively.

### DNA manipulation

The primers used for plasmid construction and gene knockout are listed in Additional file 1: Table S2. For gene knockout, the target genes *pgdS* (ID: 936837, encoding gamma-DL-glutamyl hydrolase), *ggt* (ID: 940001, encoding membrane bound gamma-glutamyltranspeptidase), *sucA* (GenBank: CP053102.1, encoding 2-oxoglutarate dehydrogenase E1 component), *alsS* (ID: 936852, encoding alpha-acetolactate synthase), *proB* (ID: 936790, encoding glutamate 5-kinase), *gudB* (ID: 938975, encoding cryptic glutamate dehydrogenase) and *rocG* (ID: 937066, encoding NAD-specific glutamate dehydrogenase) were first identified in the *Bacillus subtilis subsp. subtilis str.* 168 complete genome in NCBI. Related genes were identified in *B. tequilensis* BL01 by sequence alignment. The *pgdS* gene deletion was used as an example to describe the construction of the knockout plasmid. First, the gRNA of *pgdS* was obtained using online software (https://www.atum.bio/eCommerce/cas9/ input). The upstream (*pgdS*-N20-F) and downstream (*pgdS*-N20-R) primers of the gRNA were annealed and then inserted into the plasmid pBAC-Cas9 with the Golden Gate cloning method to form pBAC-Cas9-N20-*pgdS*. Then, the upstream homologous arm with the primer (*pgdS*-up-F, *pgdS*-up-R) and the downstream homologous arm with the primer (*pgdS*-down-F, *pgdS*-down-R) of the *pgdS* gene were amplified, each approximately 1000 bp. Subsequently, the two fragments were joined together using the In-Fusion cloning method. Finally, the homologous arm and the plasmid pBAC-Cas9-N20-*pgdS* were digested by the *SfiI* restriction enzyme (Thermo Scientific) and ligated to obtain the knockout plasmid pBAC-Cas9-N20-*pgdS*-up-down. The knockout plasmid was transformed into *B. tequilensis* BL01 as previously described [26]. Correct transformants were verified by colony PCR on LB plates containing 5 μg/mL chloramphenicol. The correct transformants were inoculated in LB medium containing 0.1 mM IPTG, and after 12 h of incubation, the gene knockout strains were selected.

The genes *citZ* (Gene ID: 937381, encoding citrate synthase II), *icd* (Gene ID: 938183, encoding isocitrate dehydrogenase), *gltA* (Gene ID: 940024, encoding glutamate synthase), *pgsBCAE* (GenBank: CP053102.1, encoding poly-gamma-glutamate synthase complex), *pycA* (ID: 935920, encoding pyruvate carboxylase), *pyk* (GenBank: QJR47503.10, encoding pyruvate kinase) and *pdhABCD* (GenBank: CP053102.1, encoding pyruvate dehydrogenase complex) in the type strain *B. subtilis subsp. subtilis str.* 168 complete genomes were first found in NCBI, and then related genes were amplified from *B. tequilensis* BL01 with the corresponding primers (Additional file 1: Table S2), and ligated to pP43NMK to construct the plasmids *P43*-*citZ*, *P43*-*icd*, *P43*-*gltA*, *P43*-*pgsBCAE*, *P43*-*pycA*, *P43*-*pyk*, *P43*-*pdhABCD*, *P43*-*citZ*-*icd*-*gltA*, and *P43*-*citZ*-*pyk*.

### Screening of a high γ-PGA-producing strain and phylogenetic analysis

γ-PGA-producing strains were isolated from soybean purchased from farmers’ markets in Tianjin, China, and the screening method was performed as previously described [14]. Soybeans (100 g) were boiled for 10 min in 500 mL of sterile water in a water bath to remove any non-spore strains. The solution was diluted 10^–1^, 10^–2^, 10^–3^, and 10^–4^ times after cooling, and 200 μL aliquots were placed on basal medium agar plates and incubated at 37 °C for 24–48 h. Colonies with high viscosity and mucosity were selected and cultured in flasks to examine their γ-PGA production abilities.

The strain with the highest γ-PGA production was subjected to 16S rDNA sequence analysis. The 16S rDNA gene sequence was amplified using universal 27F and 1492R primers. Sequencing was performed by GENEWIZ Inc. (Suzhou, China). The 16S rDNA gene sequence was compared with that of type strains reported in the EzBioCloud database (https://www.ezbiocloud.net/). The phylogenetic tree was reconstructed using the neighbor-joining method in MEGA 7.0 [27].

### One factor at a time (OFAT) experimentation design

The main nutritional components, including the sources of carbon and nitrogen and culture temperature, were optimized using the OFAT experimental design. To optimize the carbon sources, 30 g/L (w/v) glucose, sucrose, fructose, glycerol, arabinose, and xylose were added to the fermentation medium. To determine the effect of different nitrogen sources, 5 g/L (w/v) NH_4_Cl, peptone, tryptone, yeast extract, and (NH_4_)_2_SO_4_ were added to the medium. After optimizing carbon and nitrogen sources, the effect of temperature (28–42 °C) on bacterial growth and γ-PGA production was studied.

### Identification of the type of B. tequilensis BL01 for the production of γ-PGA

To identify the type of *B*. *tequilensis* BL01 for the production of γ-PGA, the identification medium contained 30 g/L sucrose, 0.5 g/L K_2_HPO_4_, 0.5 g/L MgSO_4_·7H_2_O, 0.04 g/L FeCl_3_·6H_2_O, 0.104 g/L MnSO_4_·H_2_O, 0.15 g/L CaCl_2_, 0.5 g/L NaCl and (1) 5 g/L yeast extract; (2) 5 g/L (NH_4_)_2_SO_4_; (3) 5 g/L monosodium glutamate; (4) 5 g/L yeast extract and 10 g/L sodium citrate; (5) 5 g/L (NH_4_)_2_SO_4_ and 10 g/L sodium citrate; and (6) 5 g/L monosodium glutamate and 10 g/L sodium citrate.

### Cultural methods for γ-PGA production

For shake-flask fermentation, a single colony from the agar plate was inoculated into a 250 mL flask containing 50 mL of liquid basal medium and cultured at 37 °C for 12 h at 200 rpm. Subsequently, 1% (v/v) of the precultures (OD_600_ = 5.0 ± 0.1) were inoculated into 250 mL flasks containing 50 mL of fresh fermentation medium and cultured at 37 °C for 48 h at 200 rpm. Cell growth and γ-PGA yield were measured every 6 h.

For fed-batch fermentation, cells were precultured in 300 mL of the basal medium at 37 °C for 12 h at 200 rpm and then inoculated in a 5 L fermenter (T&J-BType 5 L; T&J Bioengineering Co., Ltd., Shanghai, China) containing 2.7 L of fresh medium. The 5 L fermenter was operated at an aeration rate of 10 NL/min, and dissolved oxygen (DO) was kept above 5% by adjusting the agitation rate to 400–700 rpm. The pH was controlled at pH 6.5 ± 0.2 with 1 M HCl or NH_3_·H_2_O (25–28%). Sugar and citric acid levels were monitored regularly, and the consumption per hour was speculated to determine the replenishment point. When the sugar level dropped below 10 g/L, and the citric acid dropped below 5 g/L, 150 mL of feed medium containing 700 g/L sucrose and 20 g of sodium citrate dissolved in 50 mL sterile water were fed into the fermenter, individually.

### Analytical methods

Cell biomass was determined by measuring the absorbance of the fermentation broth at 600 nm using a spectrophotometer. The standard curve relating OD to cell dry weight (CDW, biomass) was used (1 OD600 = 0.414 g CDW/L). The concentration of γ-PGA was determined by a CTAB-dependent spectrophotometric assay, as previously described [14]. Sucrose, glucose, and fructose levels were determined using an Agilent 1260 high-performance liquid chromatography (HPLC) system equipped with a refractive index detector (RID) and an Aminex HPX-87P column (300 × 78 mm; Bio-Rad, Hercules, CA, USA). An Aminex HPX-87H column (300 × 78 mm; Bio-Rad, Hercules, CA, USA) was used to analyze citric acid and fermentation byproducts [14]. The γ-PGA molecular weight was measured using gel permeation chromatography (GPC) with an RID detector and an Ultrahydrogel^™^ linear column (10 µm, 7.8 mm × 300 mm, Waters Corporation, USA). The mobile phase, flow rate, and injection volume were 0.1 N NaNO_3_, 0.5 mL/min, and 20 µL, respectively. Glutamic acid was measured using an Agilent ZORBAX Eclipse Plus C18 column (5 µm, 4.6 mm × 150 mm, Agilent, USA) with a UV detector (338 nm) (https://www.agilent.com/cs/library/applications/5990-4547EN.pdf).

### Statistical analysis

For statistical analysis of cell growth and γ-PGA titers, the data set from each experiment was treated individually with the Baranyi model fitted to each model. Data from each experiment under all studied conditions were pooled and statistically analyzed using SPSS software (version 11.5), using a two-factor ANOVA, where the factors were biomass and γ-PGA titer, followed by a Tukey test, with significant differences at *P* < 0.05.

## Results and discussion

### Screening of highly γ-PGA-producing strains

To screen for a highly γ-PGA-producing strain, 24 isolates with mucoid colonies were selected from basal medium agar plates. Five strains produced γ-PGA during shake flask fermentation, and their 16S rDNA genes (1425 bp) were amplified and sequenced. BLAST analysis using EzBioCloud (https://www.ezbiocloud.net/) showed that these strains belonged to different species (Additional file 1: Table S3). BL01 produced 8.6 ± 0.3 g/L γ-PGA after 24 h of incubation, the γ-PGA titer normalized to the biomass of the individual cultures was the highest compared to other isolates, and BL01 was classified as the *B. tequilensis* species.

Fermented soybean foods such as natto and cheonggukjang contain high levels of γ-PGA [28, 29]. Therefore, soybean was selected as the screening material for γ-PGA-producing strains. Previous studies found that *B. tequilensis* can quickly and stably colonize plants, and has a high propagation rate and a strong proliferative capacity [30]. *B. tequilensis* has high efficiency and broad-spectrum resistance to plant pathogens, has a combined treatment effect on plant diseases such as anthracnose [31] and black spot disease [32], has a high prevention effect, and has good application prospects for the prevention and control of plant diseases [33]. However, it has never been reported for γ-PGA production; therefore, *B. tequilensis* BL01 was selected for subsequent experiments. BL01 colonies were creamy white, mucoid, translucent, and grown on a solid culture medium. A phylogenetic tree was generated based on 16S rDNA gene sequences, as shown in Additional file 1: Fig. S1.

### Optimization of nutritional and culture parameters for γ-PGA production

**Fig. 2:**
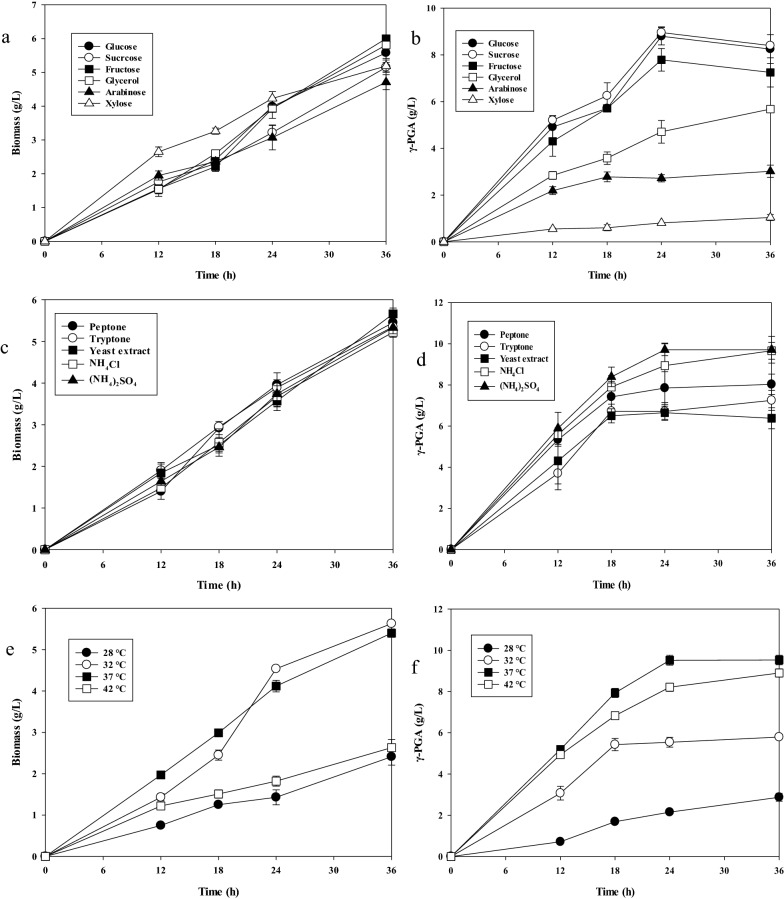
One-factor-at-a-time method for γ-PGA production by *B. tequilensis* BL01. **a** Effect of carbon sources on cell growth; **b**, Time-course γ-PGA output profile in different carbon sources. **c** Effect of nitrogen sources on cell growth; **d** Time course profile of the γ-PGA output in various nitrogen sources. **e** Effect of temperature on cell growth; **f** Time course profile of γ-PGA output at different temperatures. Values represent the mean ± SD, n = 3

All cells grew well, but after 36 h of incubation, different γ-PGA levels were obtained in a culture medium with varying sources of carbon (Fig. 2a and b). In glucose- and sucrose-based media, the γ-PGA titers gradually increased, peaking at 8.9 ± 0.2 g/L at 24 h. The γ-PGA production and cell growth rates of the two different carbon sources were approximately the same (*P* > 0.05), but the γ-PGA titer normalized to the biomass of the sucrose-based medium was higher than that of the medium with glucose as the carbon source (Additional file 1: Fig. S2a). Furthermore, the highest biomass (6.0 ± 0.1 g/L) was obtained in the fructose medium, which showed an asymmetric relationship with the γ-PGA level (7.8 ± 0.4 g/L). Small amounts of γ-PGA were produced from the other carbon sources in the order of glycerol (5.6 ± 0.1 g/L) > arabinose (3.0 ± 0.2 g/L) > xylose (1.1 ± 0.1 g/L) (*P* < 0.05) (Fig. 2a). Carbon source metabolism at the γ-PGA level is related to several stress response proteins, such as catabolite control protein A (CcpA) [34]. Fructose bisphosphate and excess glucose activate CcpA [35]. In *B. licheniformis* and *B. subtilis*, CcpA directly or indirectly regulates the expression of *pgsB*, which encodes PGA synthetase [36]. PGA synthetase is more likely to be strongly expressed in sucrose-, glucose-, and fructose-based media than in media made with other carbon sources. Furthermore, the conversion of glutamic acid to 2-oxoglutarate is negatively controlled by CcpA [34]. This could explain the various γ-PGA production in media with different carbon sources. In *B. subtilis* NX-2, glycerol improves γ-PGA production by reducing viscosity and increasing substrate uptake during fermentation [12]. However, in this study, the highest γ-PGA titer was not obtained from the glycerol-based medium, which is consistent with *B. siamensis* [14]. When glycerol is used as the substrate, the pentose phosphate pathway (PPP) may be weakened to cause a shortage of NADPH, and the shortage of NADPH may be a reason for the insufficient production of γ-PGA [37].

Experiments on the effect of nitrogen sources on cell growth and γ-PGA production were conducted in sucrose-based medium. As shown in Fig. 2c and d, all nitrogen sources supported cell growth and γ-PGA production. The γ-PGA titer of the medium with yeast extract as a nitrogen source was the lowest (6.4 ± 0.5 g/L), and that of the medium with ammonium sulfate as a nitrogen source was the highest at 9.7 ± 0.3 g/L. In addition, the γ-PGA titer normalized to the biomass was highest in the medium with ammonium sulfate as the nitrogen source (Additional file 1: Fig. S2b). Additionally, there were no significant differences in cell growth rates between all tested nitrogen sources (*P* > 0.05). In general, γ-PGA production was higher with inorganic nitrogen sources than with organic nitrogen sources, but organic nitrogen sources promoted cell growth, consistent with several previous reports [1]. Under the action of NADPH-dependent glutamate dehydrogenase (GDH), free NH_4_^+^ reacts with α-ketoglutarate in the tricarboxylic acid cycle to form glutamate, which contributes to γ-PGA production [17, 18].

Figure 2e and f show that γ-PGA production and cell growth were significantly affected by culture temperature. The highest γ-PGA titer (9.5 ± 0.2 g/L) with a biomass of 5.4 ± 0.1 g/L was obtained at 37 °C. Although the highest biomass (5.6 ± 0.1 g/L) was obtained at 32 °C, the γ-PGA titer was only 5.8 ± 0.2 g/L. Higher temperature (42 ℃) and lower temperature (28 ℃) were detrimental to cell growth. Although 8.9 ± 0.2 g/L γ-PGA was obtained at 42 °C, the γ-PGA titer normalized to the biomass was the highest compared to other temperatures (Additional file 1: Fig. S2c). This may be due to the increased flux from isocitrate to 2-oxoglutarate and from 2-oxoglutarate to glutamate at higher temperatures [38]. Temperature mainly affects enzyme activity, and for most isolated *Bacillus* strains, the optimal temperature for γ-PGA production is approximately 37 °C [14, 39].

### Identification of the type of *B. tequilensis* BL01 for the production of γ-PGA

**Fig. 3:**
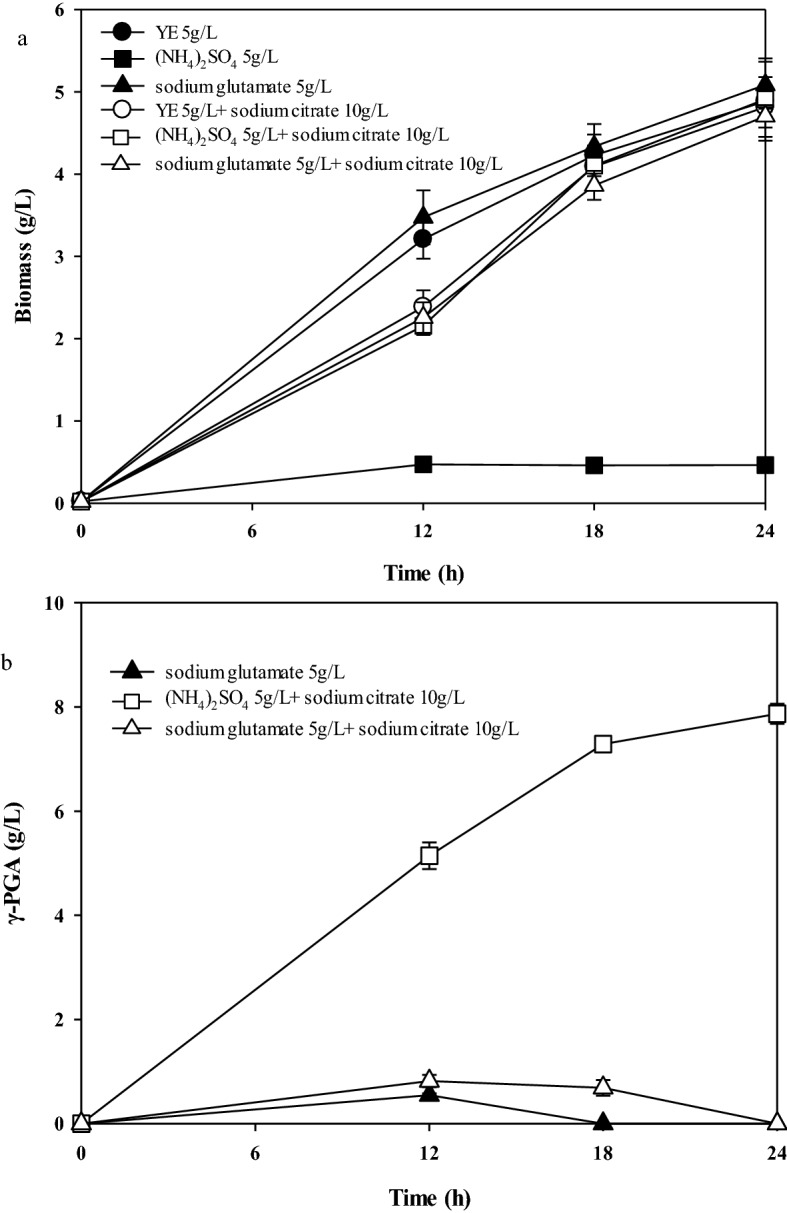
Identify the type of *B. tequilensis* BL01 for producing of γ-PGA by adding different nitrogen sources to the culture medium. **a** Cell growth curves, **b** γ-PGA growth curves. Values represent the mean ± SD, n = 3

γ-PGA producing strains are divided into glutamic acid-dependent and glutamic acid-independent strains. For glutamic acid-independent strains, the carbon source can synthesize γ-PGA directly [16, 18, 20]. Additionally, citric acid can be used as a direct precursor to improve γ-PGA production [17]. To identify the γ-PGA production characteristic of *B*. *tequilensis* BL01, the strain was inoculated in the medium by adding yeast extract, (NH_4_)_2_SO_4_, sodium citrate, or sodium glutamate as a nitrogen source to test cell growth and γ-PGA production. As shown in Fig. 3a, cell growth was inhibited in the medium with ammonium sulfate as the sole nitrogen source, and inhibition was alleviated by adding sodium citrate. The highest biomass (5.1 ± 0.3 g/L) was obtained by adding sodium glutamate to the medium. The highest titer of γ-PGA (7.8 ± 0.2 g/L) was obtained by adding sodium citrate and (NH_4_)_2_SO_4_ to the medium. No γ-PGA was produced by adding only (NH_4_)_2_SO_4_ and yeast extract to the medium. When 5 g/L sodium glutamate was added, 0.6 ± 0.1 g/L γ-PGA was produced in 12 h, which was then rapidly consumed (Fig. 3b). The results showed that the organic nitrogen source could only maintain cell growth. However, the simultaneous presence of sodium citrate and (NH_4_)_2_SO_4_ in the medium not only maintains cell growth, but also promotes γ-PGA synthesis.

Free NH_4_^+^ and α-ketoglutaric acid are converted to glutamic acid by the action of aminotransferase [18, 23]. Glutamic acid performs two functions: one part of glutamic acid is degraded by GDH for nitrogen metabolism [40], and the other is converted to γ-PGA by γ-PGA synthetases (*pgsBCAE*) [23]. In this study, citric acid promoted the tricarboxylic acid cycle, producing more α-ketoglutaric acid, combined with NH4^+^ in the medium to produce glutamic acid. The results showed that *B*. *tequilensis* BL01 was a glutamic acid-independent strain for γ-PGA production. The γ-PGA produced in sodium glutamate medium decreased after 12 h, which is consistent with the idea that PGA depolymerase exists in *Bacillus spp.* [20]. Yeast extract does not produce γ-PGA. When sodium citrate was added, the same result still occurred. This is likely due to insufficient NH_4_^+^, resulting in insufficient synthesis of glutamate. Therefore, γ-PGA cannot be produced.

### Disruption of pgdS and ggt genes in the *B. tequilensis* BL01 strain

**Fig. 4:**
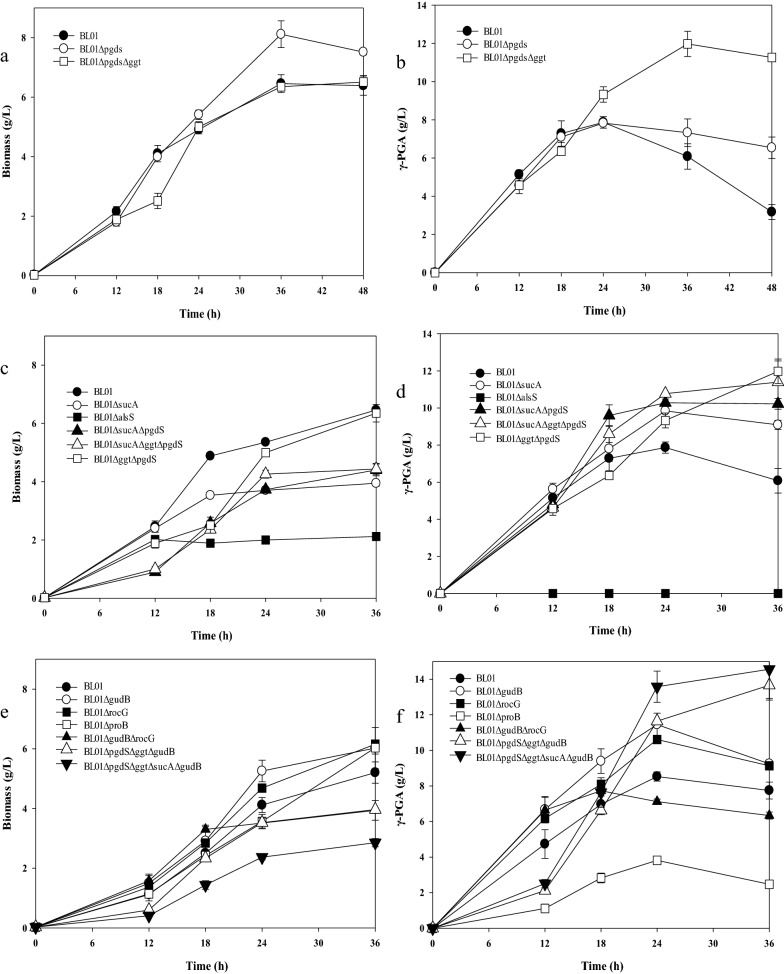
Effect of the disruption of the *pgdS* and *ggt* genes in the strain of *B. tequilensis* BL01, **a** Cell growth curves, **b** γ-PGA growth curves; Effect of the disruption of the *alsS* and *sucA* genes, **c** Cell growth curves, **d** γ-PGA growth curves; Effect of regulating the level of glutamic acid on γ-PGA synthesis, **e** Cell growth curves, **f** γ-PGA growth curves. Values represent the mean ± SD, n = 3

To decrease the degradation rate and further improve the γ-PGA production of *B*. *tequilensis* BL01, the PGA depolymerase genes, including gamma-DL-glutamyl hydrolase (*pgdS*) and membrane-bound gamma-glutamyltranspeptidase (*ggt*) were deleted to obtain *B*. *tequilensis* BL01Δ*pgdS* and the double deletion strain *B*. *tequilensis* BL01Δ*pgdS*Δ*ggt*. The growth profiles and γ-PGA production of the mutants and the wild-type strain were tested in medium supplemented with sodium citrate, as shown in Fig. 4a and b. The mutant strains presented a growth pattern similar to the wild-type strain. The highest γ-PGA production was found in *B*. *tequilensis* BL01Δ*pgdS*Δ*ggt*, yielding 11.9 ± 0.6 g/L γ-PGA compared with 7.8 ± 0.3 g/L from the wild type (a 52.6% increase). *B*. *tequilensis* BL01Δ*pgdS* showed γ-PGA production similar to that of the wild-type strain. Furthermore, it was found that after the knockout of *pgdS* and double deletion of *pgdS* and the *ggt* gene, the decomposition rate of γ-PGA was significantly reduced by prolonging the culture time. For all strains, lactic acid was consumed at the later stage of fermentation (Additional file 1: Fig. S3a), and a small amount of acetic acid accumulated (Additional file 1: Fig. S3b). The main byproduct was 2, 3-BD (Additional file 1: Fig. S3c).

Knockout of γ-PGA degrading enzyme genes to improve γ-PGA production has been extensively studied. Single-gene knockout of the *pgdS* or *ggt* gene did not significantly improve γ-PGA production in *B. subtilis* 168, while simultaneous knockout of the *pgdS* and *ggt* genes doubled the yield of γ-PGA [41]. Our results are the same as those of this study. Deletion of the two γ-PGA-degrading enzyme genes, a higher γ-PGA yield, and a reduced γ-PGA degradation rate were obtained. Although these results are very encouraging, more in-depth research is needed in the future on the interaction of γ-PGA-degrading enzyme changes in the expression levels of other genes associated with γ-PGA synthesis and degradation, and the metabolism of the carbon and nitrogen source.

### Disruption of alsS and sucA genes in the *B. tequilensis* BL01 strain

Although the titer of γ-PGA synthesized by *B*. *tequilensis* BL01Δ*pgdS*Δ*ggt* was improved, the main byproduct 2,3-BD was contained, which decreased the substrate conversion rate of sucrose to γ-PGA. In addition, a sufficient precursor is also very important for the efficient accumulation of γ-PGA. Therefore, alpha-acetolactate synthase (*alsS*) and the 2-oxoglutarate dehydrogenase E1 component (*sucA*) were deleted to block the 2,3-BD synthesis pathway and tricarboxylic acid cycle (TCA). It was found that deletion of the *alsS* gene was not conducive to strain growth, and that there was no γ-PGA production. The titer of acetic acid was increased by 20% compared to the wild-type strain and became the main byproduct (Additional file 1: Fig. S4a). The amount of lactic acid was maintained at a low level (Additional file 1: Fig. S4b). The γ-PGA yield of *B*. *tequilensis* BL01Δ*sucA* was approximately 1.25-fold (9.8 ± 0.4 g/L *v* 7.8 ± 0.3 g/L) higher than that of BL01, while cell growth was slower than that of BL01. Furthermore, the double deletion strain *B*. *tequilensis* BL01Δ*sucA*Δ*pgdS* and the triple deletion strain *B*. *tequilensis* BL01Δ*sucA*Δ*pgdS*Δ*ggt* produced more γ-PGA, and the titers reached 10.3 ± 0.2 g/L and 11.4 ± 0.7 g/L, respectively (Fig. 4c, d).

The *alsS* gene encodes α-acetolactate synthase, which condenses two molecules of pyruvate to form acetolactate. Acetolactate is converted to acetoin by the action of acetolactate decarboxylase, followed by the synthesis of 2,3-BD [42]. In *B. subtilis*, after disruption of the *alsS* gene, the strain does not grow unless valine or isoleucine is added. In addition, blockade of the 2,3-BD metabolic pathways causes a deficiency in NAD^+^ pools [43]. During γ-PGA synthesis, α-ketoglutarate binds to ammonia and produces L-glutamate acid, which requires NADPH and releases NADP^+^ [22]. For *B*. *tequilensis* BL01, the effect of *alsS* gene knockout may be due to the NADH/NAD^+^ imbalance. The *sucA* gene (encoding α-ketodehydrogenase) was deleted to block the TCA cycle; thus, cell growth was lower than that of the wild-type strain, but the reduced degradation of α-ketoglutarate increased the γ-PGA titer. Interestingly, the growth rate of γ-PGA was much higher in the *B*. *tequilensis* BL01Δ*sucA*Δ*pgdS*Δ*ggt* strain than in the *B*. *tequilensis* BL01Δ*pgdS*Δ*ggt* strain before 24 h (Fig. 4d). After 24 h, the cells of the triple knockout strain almost stopped growing, which decelerates γ-PGA production (Fig. 4c, d). This may be caused by environmental stress and metabolic deficiencies (blocked TCA). Finally, there was no significant difference in γ-PGA titers between the *B*. *tequilensis* BL01Δ*sucA*Δ*pgdS*Δ*ggt* and *B*. *tequilensis* BL01Δ*pgdS*Δ*ggt* strains (11.4 ± 0.7 g/L *vs* 11.9 ± 0.6 g/L) (Fig. 4d).

### Effect of regulating the level of glutamic acid on γ-PGA synthesis

Glutamate is the precursor for γ-PGA synthesis; the accumulation of glutamate through the regulation of pathways seems to be a way to improve the γ-PGA titer. Glutamate 5-kinase (*proB*), cryptic glutamate dehydrogenase (*gudB*), and NAD-specific glutamate dehydrogenase (*rocG*) in *B*. *tequilensis* BL01 were removed to construct strains *B*. *tequilensis* BL01Δ*proB*, *B*. *tequilensis* BL01Δ*gudB* and *B*. *tequilensis* BL01Δ*rocG*. When glutamate degradation genes were knocked out, there was a significant increase in glutamic acid production compared to the control (Additional file 1: Fig. S5). Knockout of *gudB* and *rocG* increased cell growth (Fig. 4e). Furthermore, the γ-PGA titers of the two strains were also improved by 33.6% and 24.4% compared to the wild-type strain of *B*. *tequilensis* BL01, respectively (Fig. 4f). However, knockout of the *proB* gene not only inhibited cell growth but also reduced the γ-PGA titer. Then, *gudB* and *rocG* were knocked out together in *B*. *tequilensis* BL01 to obtain strain *B*. *tequilensis* BL01Δ*gudB*Δ*rocG*. Unfortunately, the biomass and γ-PGA titer decreased to 3.5 ± 0.1 g/L and 7.1 ± 0.1 g/L, respectively. Based on the above results (Figs. 4b, d), we constructed multigene knockout strains *B*. *tequilensis* BL01Δ*pgdS*Δ*ggt*Δ*gudB* and *B*. *tequilensis* BL01Δ*pgdS*Δ*ggt*Δ*sucA* Δ*gudB*. The two strains showed increases of 20.2% and 28.1% in γ-PGA titers (13.7 ± 0.8 g/L & 14.6 ± 0.6 g/L) compared to the strain *B*. *tequilensis* BL01Δ*gudB* (11.4 ± 0.6 g/L) (Fig. 4f). Although γ-PGA increased, cell growth in the two strains (3.9 ± 0.1 g/L and 2.8 ± 0.1 g/L) was reduced compared to that in the control (6.0 ± 0.7 g/L) (Fig. 4e). The results showed that biomass formation decreases when γ-PGA production increases. One reason may be due to the insufficient nitrogen source (glutamate) affecting cell growth, since γ-PGA synthesis competes with cell growth for carbon and nitrogen sources. The other is that inadequate dissolved oxygen reduces cellular metabolism due to the accumulation of γ-PGA.

*RocG* is a bifunctional enzyme, and its activity is strain dependent. In *B. licheniformis*, the glutamate synthesis rate was much higher than degradation under the catalysis of *rocG* [44, 45]. However, in *B. subtilis*, *rocG* performs two functions. The first is devoted to glutamate, which forms 2-oxoglutarate. Another role is to inhibit *GltC*, which activates glutamate synthase (*gltAB*) [25, 46]. In our study, the activity of *rocG* was more prone to glutamate degradation. Therefore, the deletion of *rocG* can activate glutamate synthesis and improve the titer of γ-PGA. The *gudB* gene, which harbors an insertion of three amino acids, encodes an inactive glutamate dehydrogenase in *B. subtilis* [47]. However, in *B*. *tequilensis* BL01, the insertion of three amino acids does not exist, suggesting that glutamate degradation occurs. The *gudB* mutant strain presented a higher γ-PGA production than *B*. *tequilensis* BL01Δ*rocG*, and *gudB* may play a more important role than *rocG*. The *gudB* and *rocG* double mutant strain presented lower γ-PGA production than the *gudB* or *rocG* mutant strains, possibly caused by the obvious growth defect in the fermentation medium (Fig. 4e). Proline serves as an important high osmotic pressure and butanol chaotropic stress protectant for *B. subtilis* [48, 49]. In *B*. *tequilensis* BL01Δ*proB*, the proline synthesis pathway was blocked, and the strain could not resist the high osmotic pressure caused by the accumulation of γ-PGA and 2,3-BD, resulting in limited growth.

### Effect of gene overexpression on γ-PGA synthesis

Accumulating more precursors (citric acid and glutamic acid) is effective in increasing γ-PGA production. To improve the supply of precursors, in addition to reducing glutamate consumption (deletion of the *gudB*, *rocG* and *proB* genes), another way is to obtain sufficient precursors through enhanced metabolism. α-Ketoglutaric acid is an important intermediate metabolite in the TCA cycle, and glutamic acid is generated from α-ketoglutaric acid. However, a large amount of byproducts (lactic acid, acetic acid and 2,3-BD et al*.*) are converted from pyruvate, which reduces the metabolic flow of carbon sources to glutamate [23]. Therefore, seven genes from the TCA pathway and γ-PGA synthesis pathway were overexpressed in *B*. *tequilensis* BL01, including pyruvate dehydrogenase (*pdhABCD*), pyruvate carboxylase (*pycA*), pyruvate kinase (*pyk*), citrate synthase (*citZ*), isocitrate dehydrogenase (*icd*), glutamate synthase (*gltA*) and polyglutamate synthase (*pgsBCAE*).

**Fig. 5:**
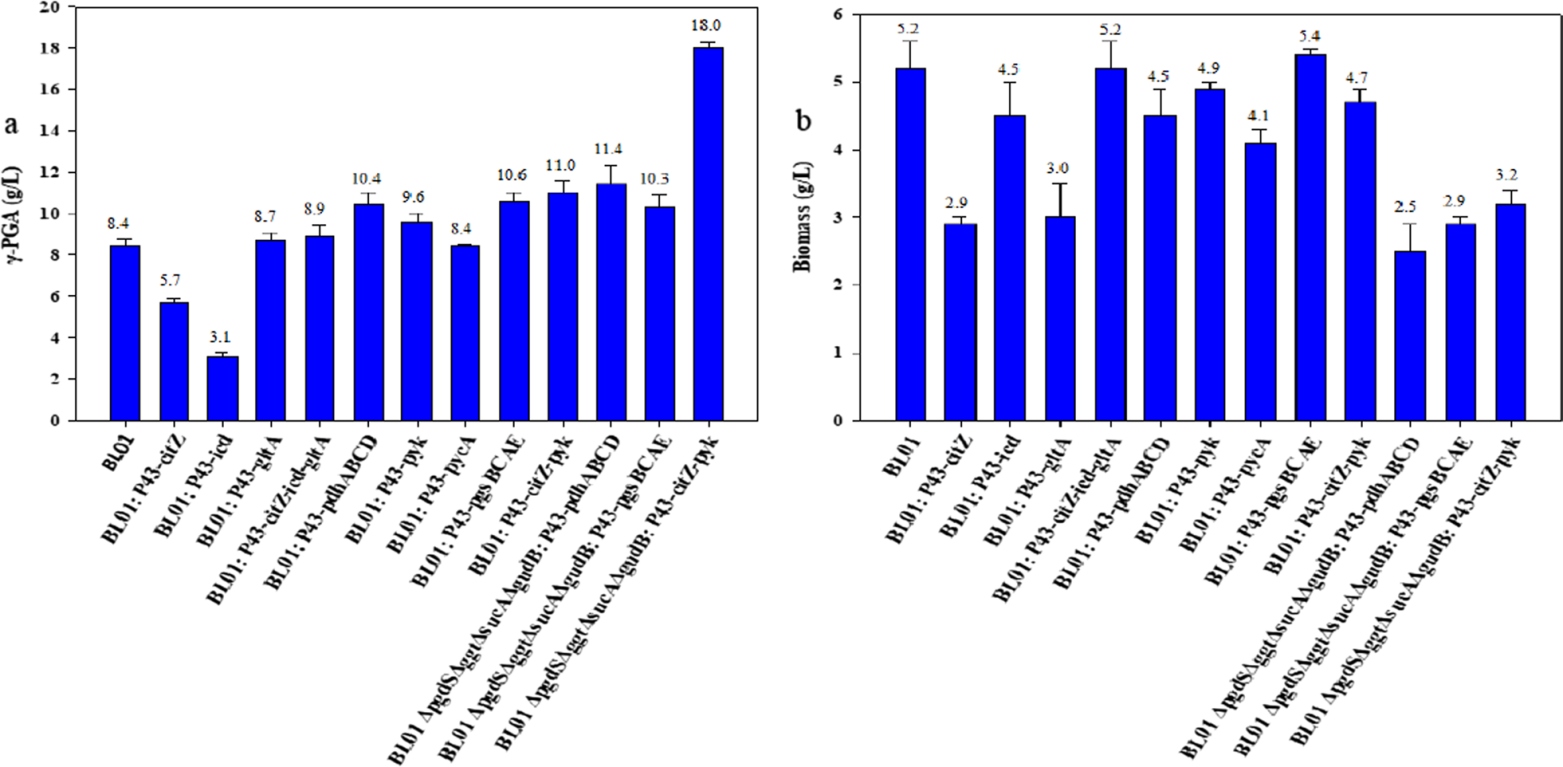
Effect of overexpression of genes for γ-PGA production in *B. tequilensis* BL01 and mutant strains at 24 h, **a** γ-PGA titers, **b** Cell growth. Data are given as the mean ± SD, n = 3

First, we overexpressed the *citZ*, *icd* and *gltA* genes, resulting in the recombinant strains BL01:*P43*-*citZ*, BL01:*P43*-*icd*, BL01:*P43*-*gltA*, and BL01:*P43*-*citZ*-*icd*-*gltA*. Contrary to our predictions, the γ-PGA titers were not enhanced in these recombinant strains (Fig. 5a). We hypothesize that the rate-limiting step for glutamic acid synthesis may come from upstream of the TCA cycle. Therefore, the *pdhABCD*, *pycA* and *pyk* genes as well as the *pgsBCAE* gene were overexpressed to test γ-PGA production. The overexpression of the *pdhABCD*, *pyk*, and *pgsBCAE* genes was found to significantly increase the accumulation of γ-PGA, and the γ-PGA titers of the engineered strains reached 10.4 ± 0.6, 9.6 ± 0.4 and 10.6 ± 0.4 g/L, respectively (Fig. 5a). Although the overexpression of the *citZ* gene reduced γ-PGA production, when cultured in medium without the addition of citric acid, the *citZ* gene overexpression strain showed an excellent growth advantage compared to the control strain (Additional file 1: Fig. S6). This suggests that overexpression of the *citZ* gene enables more carbon source to flow toward citric acid synthesis. Therefore, the *citZ* and *pyk* genes were coexpressed to verify γ-PGA production. The γ-PGA titer of BL01:*P43*-*citZ*-*pyk* was 11.0 ± 0.6 g/L. We have also tried to co-overexpress other genes (such as *citZ* and *pdhABCD*; *pdhABCD* and *pyk*; *pyk* and *pgsBCAE *et al*.*), but transformation into *B*. *tequilensis* BL01 always failed (data not shown). In addition, the overexpression plasmids with significant effects on γ-PGA production were transferred to *B*. *tequilensis* BL01Δ*pgdS*Δ*ggt*Δ*sucA*Δ*gudB* to obtain different engineered strains. The γ-PGA production of strain *B*. *tequilensis* BL01Δ*pgdS*Δ*ggt*Δ*sucA*Δ*gudB*:*P43*-*citZ*-*pyk* was increased to 18.0 ± 0.3 g/L, increase of 23.3% compared to that of the host strain (14.6 ± 0.6 g/L). Unfortunately, overexpression of the *pdhABCD* and *pgsBCAE* genes in *B*. *tequilensis* BL01Δ*pgdS*Δ*ggt*Δ*sucA*Δ*gudB* reduced γ-PGA production, and the γ-PGA titers obtained in the two engineered strains were only 11.4 ± 0.9 g/L and 10.3 ± 0.4 g/L, respectively (Fig. 5a).

Previous studies have reported that improving the precursor supply plays a vital role in γ-PGA synthesis. Zhu et al. overexpressed the *citZ*, *icd*, and *gltA* genes in *B. amyloliquefaciens*, which improved the synthesis of γ-PGA from crude glycerol [24]. Li et al. overexpressed the pyruvate dehydrogenase (*pdhABCD*) gene, resulting in a 34.93% increase in the titer of γ-PGA in *B. licheniformis* WX-02 [23]. In our study, only overexpression of the *pdhABCD* gene in *B*. *tequilensis* BL01 showed positive results for γ-PGA synthesis. This suggests that for enhanced precursor synthesis, the rate-limiting step is reflected in the direction of pyruvate metabolism toward the branch of the TCA cycle (Fig. 1). We also tried to co-overexpress the *citZ* and *pdhABCD* genes and other combinations, but transformation into *B. tequilensis* BL01 always failed. We believe that the enhanced metabolic pathway of pyruvate toward the tricarboxylic acid cycle affects cell growth and can even lead to lethality. This effect is equivalent to knocking out the *alsS* gene, which inhibits cell growth (Figs. 4c, 5b). In contrast to our speculation, the overexpression of *pdhABCD* and *pgsBCAE* did not lead to an increase in γ-PGA production compared to *B*. *tequilensis* BL01Δ*pgdS*Δ*ggt*Δ*sucA*Δ*gudB*. The genetically engineered BL01 strain might disrupt cell balance or membrane-associated metabolic activity, resulting in reduced γ-PGA production [20].

### Fed-batch fermentation for γ-PGA production

**Fig. 6:**
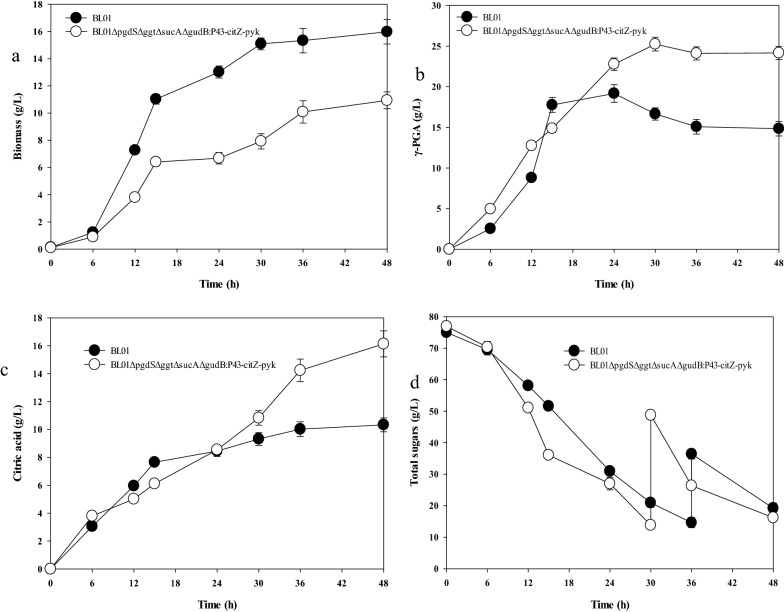
Time curves of fed-batch fermentation of γ-PGA in a 5 L fermenter by BL01 and BL01Δ*pgdS*Δ*ggt*Δ*sucA*Δ*gudB*:*P43*-*citZ*-*pyk* strain, **a** Cell growth curves, **b** γ-PGA growth curves, **c** Citric acid consumption curves, **d** Residual sugar curves. Data are given as the mean ± SD, n = 3. Aeration rate: 10 mL/min; agitation rate: 400 rpm (0–6 h), 500 rpm (6–12 h), 600 rpm (12–24 h), 700 rpm (24–48 h); the pH was controlled at pH 6.5 ± 0.2. 20 g of sodium citrate dissolved in 50 mL of sterile water was fed into the fermenter at 24 h, and 150 mL of feed medium containing 700 g/L of sucrose was fed at 30 and 36 h, individually

To further increase the level of γ-PGA using BL01 and the engineered strain, a scale-up experiment was carried out on a 5 L fermenter by initially adding 10 g/L sodium citrate to the culture medium. As shown in Fig. 6a, biomasses of 16.0 ± 0.9 g/L and 10.9 ± 0.6 g/L in BL01 and the engineered strain were achieved after 48 h of cultivation. The results of the fed-batch fermentation of BL01Δ*pgdS*Δ*ggt*Δ*sucA* Δ*gudB*:*P43*-*citZ*-*pyk* showed that a γ-PGA titer of 25.3 ± 0.8 g/L was obtained, an increase of 32.4% compared to the BL01 strain (19.2 ± 1.1 g/L). The highest productivity of γ-PGA reached 0.84 g/L/h after 30 h of cultivation (Fig. 6b). The citric acid conversion rate of γ-PGA synthesized by the wild-type and engineered strains reached 1.44 and 1.50 g/g (g-γ-PGA/g-citric acid), respectively (Fig. 6c). 2,3-BD was the main byproduct; although the engineered strain increased the rate of sugar consumption (Fig. 6d), the 2,3-BD titer was lower (Additional file 1: Fig. S7a) but increased the acetic acid titer compared to the BL01 strain (Additional file 1: Fig. S7b). The γ-PGA molecular weights of BL01 and the engineered strain were 1.84 × 10^5^ Da (Additional file 1: Fig. S8a) and 2.06 × 10^6^ Da (Additional file 1: Fig. S8b), respectively. The results suggest that depending on the application of the γ-PGA, either BL01 or the engineered strains can be used to selectively produce γ-PGA of low (1.84 × 10^5^ Da) or high MW (2.06 × 10^6^ Da), respectively.

In this study, the “push-pull-inhibit” strategy was applied to engineer the *B. tequilensis* BL01 strain: deleting the γ-PGA degrading enzyme genes, reduces glutamate consumption and enhances precursors generation. Although the reported metabolic engineering strategies were already employed elsewhere alone, there can be some variation in the results, probably due to the differences in strains [25, 41, 44, 45]. This study first demonstrated a system engineering strategy to enhance γ-PGA synthesis in *B. tequilensis* BL01, and the finally obtained BL01Δ*pgdS*Δ*ggt*Δ*sucA* Δ*gudB*:*P43*-*citZ*-*pyk* strain could produce 25.2 g/L γ-PGA in a 5 L fermenter. During fed-batch fermentation, the production of γ-PGA increases the viscosity of the medium and thus reduces dissolved oxygen and mass transport. Although the remaining high dissolved oxygen (≥ 20%) was beneficial to improve γ-PGA production [17], a further increase in the agitation speed with the highest aeration only maintained the dissolved oxygen below 5%, which is the minimum dissolved oxygen value for the production of γ-PGA [17]. Dissolved oxygen directly affects cell growth, γ-PGA titer, and productivity, and is an essential parameter of fermentation control. Aerobic respiration occurs with the appropriate amounts of dissolved oxygen, allowing *B. tequilensis* BL01 to produce more energy, accelerating growth and γ-PGA synthesis. It is not feasible to maintain high dissolved oxygen by fermentation alone; future research will employ metabolic engineering strategies to improve dissolved oxygen, such as the expression of *Vitreoscilla* hemoglobin (*VHb*) in *B. tequilensis* BL01 [50].

*B. tequilensis* BL01 is a glutamic acid-independent strain for γ-PGA production. However, the strain itself cannot synthesize more citric acid to sustain cell growth and γ-PGA synthesis, and we must add a small amount of citric acid as a precursor. Interestingly, the yield reached 1.50 g _γ-PGA_/g _citric acid_, because citric acid can be converted from sucrose through the tricarboxylic acid cycle [23]. Future research focused on enhancing endogenous glutamate production without the exogenous addition of citric acid is necessary. The molecular weight of γ-PGA is typically 1 × 10^4^–10 × 10^6^ Da [51]. Low-molecular-weight γ-PGA (< 3 × 10^5^ Da) is typically used in food, cosmetics, and agriculture [2, 4, 11]. γ-PGA with a high molecular weight (1–2 × 10^6^ Da) is helpful for mediating antitumor immunity and may be a promising candidate for cancer immunotherapy [52]. Composite materials containing high-molecular-weight γ-PGA (1 × 10^6^ Da) have critical applications as bioadhesive hydrogels [9]. Here, we obtained different molecular weights of γ-PGA to increase the range of industrial applications.

## Conclusions

This work constructed a strain of *B. tequilensis* BL01 for the efficient synthesis of γ-PGA through a systematic genetic engineering strategy. First, the culture conditions were optimized to improve γ-PGA production. Then genes related to the glutamate synthesis pathway were overexpressed or removed to systematically optimize the metabolic network of *B. tequilensis* BL01 in the synthesis of γ-PGA. The final obtained strain BL01Δ*pgdS*Δ*ggt*Δ*sucA*Δ*gudB*:*P43*-*citZ*-*pyk* could produce 18.0 ± 0.3 g/L and 25.3 ± 0.8 g/L γ-PGA in the flask and 5 L fermenter, respectively, which was 2.17-fold and 1.32-fold higher than that of the strain *B. tequilensis* BL01 strain. The molecular weight of γ-PGA (2.06 × 10^6^ Da) produced by the mutant strain was more than 10 times higher than that produced by the wild-type strain (1.84 × 10^5^ Da). These strategies have significantly increased the titer of γ-PGA, and more work must be done to improve the γ-PGA economy.

## Supplementary Information

**Additional file 1: Figure S1.** Neighbor-joining tree based on the 16S rDNA gene sequence showing relationships between BL01 and *Bacillus* species. Bootstrap values greater than 50 are indicated at the branch nodes. Bar, 0.005 substitutions per nucleotide position. T, representative type strain. GenBank accession numbers of 16S rDNA appear in brackets. **Figure S2.** The γ-PGA titer normalized to the biomass of the individual cultures. (a) Effect of carbon sources; (b) Effect of nitrogen sources; (c) Effect of temperature. Values represent the mean ± SD, n=3. **Figure S3.** Effect of disruption of *pgdS* and *ggt* genes in the *B. tequilensis* BL01 strain. (a) Change curves of lactic acid with fermentation time, (b) Change curves of acetic acid with fermentation time, (c) Growth curves of 2,3-BD with fermentation time. Values represent the mean ± SD, n=3. **Figure S4.** Effect of disruption of *sucA* and *alsS* genes in the *B. tequilensis* BL01 strain. (a) Change curves of acetic acid with fermentation time, (b) Change curves of lactic acid with fermentation time. Values represent the mean ± SD, n=3. **Figure S5.** The glutamic acid curve of *B. tequilensis* BL01 and mutant strains. Values represent the mean ± SD, n=3. **Figure S6.** The growth curve of *B. tequilensis* BL01 and engineered strains in the medium without citric acid. Values represent the mean ± SD, n=3. **Figure S7.** Time curves of fed-batch fermentation of γ-PGA in a 5 L fermenter by BL01 and BL01Δ*pgdS*Δ*ggt*Δ*sucA*Δ*gudB*:*P43*-*citZ*-*pyk* strain, (a) 2,3-BD growth curves, (b) acetic acid growth curves. Data are given as the mean ± SD, n=3. Aeration rate: 10 mL/min; agitation rate: 400 rpm (0–6 h), 500 rpm (6–12 h), 600 rpm (12–24 h), 700 rpm (24–48 h); the pH was controlled at pH 6.5±0.2. 20g of sodium citrate dissolved in 50 mL sterile water was fed into the fermenter at 24h, and 150 mL of feed medium containing 700 g/L of sucrose was fed at 30 and 36 h, individually. **Figure S8.** The molecular weight of γ-PGA produced by (a) *B. tequilensis* BL01 and (b) *B. tequilensis* BL01Δ*pgdS*Δ*ggt*Δ*sucA*Δ*gudB*:*P43*-*citZ*-*pyk* strain. **Table S1**. Strains and plasmids used in this study. **Table S2.** Primers used in this study. **Table S3.** γ-PGA production by the isolated strains.

## Data Availability

All data generated or analyzed during this study are included in this article.

## Abbreviations

γ-PGA: Poly-γ-glutamic acid
*B. tequilensis*: *Bacillus tequilensis*
*pdhABCD*: Pyruvate dehydrogenase
*pyk*: Pyruvate kinase
*pycA*: Pyruvate carboxylase
*citZ*: Citrate synthase II
*icd*: Isocitrate dehydrogenase
*gltA*: Glutamate synthase
*pgsBCAE*: Poly-gamma-glutamate synthase complex
*alsS*: Acetolactate synthase
*rocG*: Glutamate dehydrogenase
*gudB*: Cryptic glutamate dehydrogenase
*proB*: Glutamate 5-kinase
*sucA*: 2-Oxoglutarate dehydrogenase E1 component
*pgdS*: Gamma-DL-glutamyl hydrolase
*ggt*: Membrane bound gamma-glutamyltranspeptidase
HPLC: High-performance liquid chromatography
GPC: Gel permeation chromatogram
RID: Refractive index detector

## References

[1] Luo Z, Guo Y, Liu J, Qiu H, Zhao M, Zou W, Li S (2016). Microbial synthesis of poly-gamma-glutamic acid: current progress, challenges, and future perspectives. Biotechnol Biofuels.

[2] Yu H, Liu H, Wang L, Zhang Y, Tian H, Ma X (2018). Effect of poly-γ-glutamic acid on the stability of set yoghurts. J Food Sci Technol.

[3] Khalil IR, Burns AT, Radecka I, Kowalczuk M, Khalaf T, Adamus G, Johnston B, Khechara MP (2017). Bacterial-derived polymer poly-γ-glutamic acid (γ-PGA)-based micro/nanoparticles as a delivery system for antimicrobials and other biomedical applications. Int J Mol Sci.

[4] Wang R, Wang X, Zhan Y, Xu Z, Feng X, Li S, Xu H (2019). A dual network hydrogel sunscreen based on poly-γ-glutamic acid/tannic acid demonstrates excellent anti-UV, self-recovery, and skin-integration capacities. ACS Appl Mater.

[5] Peng Y, Chang Y, Chen K, Wang C (2020). A field pilot-scale study on heavy metal-contaminated soil washing by using an environmentally friendly agent–poly-γ-glutamic acid (γ-PGA). Environ Sci Pollut Res.

[6] Chen L, Su W, Xiao J, Zhang C, Zheng J, Zhang F (2021). Poly-γ-glutamic acid bioproduct improves the coastal saline soil mainly by assisting nitrogen conservation during salt-leaching process. Environ Sci Pollut Res.

[7] Ajayeoba TA, Dula S, Ijabadeniyi OA (2019). Properties of poly-γ-glutamic acid producing-*Bacillus* species isolated from *ogi* liquor and lemon-*ogi* liquor. Front Microbiol.

[8] Wang L, Chen S, Yu B (2022). Poly-γ-glutamic acid: recent achievements, diverse applications and future perspectives. Trends Food Sci Tech.

[9] Kim MH, Lee J, Lee JN, Lee H, Park WH (2021). Mussel-inspired poly(γ-glutamic acid)/nanosilicate composite hydrogels with enhanced mechanical properties, tissue adhesive properties, and skin tissue regeneration. Acta Biomater.

[10] Xiao H, Qiu J, Wu S, Xie L, Zhou W, Wei X, Hui KN, Lin Z (2022). Cross-linked γ-polyglutamic acid as an aqueous SiOx anode binder for long-term lithium-ion batteries. ACS Appl Mater.

[11] Ma H, Li P, Liu X, Li C, Zhang S, Wang X, Tao X (2022). Poly-γ-glutamic acid enhanced the drought resistance of maize by improving photosynthesis and affecting the rhizosphere microbial community. BMC Plant Biol.

[12] Wu Q, Xu H, Liang J, Yao J (2010). Contribution of glycerol on production of poly(γ-glutamic acid) in *Bacillus subtilis* NX-2. Appl Biochem Biotech.

[13] Zhu F, Cai J, Zheng Q, Zhu X, Cen P, Xu Z (2014). A novel approach for poly-gamma-glutamic acid production using xylose and corncob fibers hydrolysate in *Bacillus subtillis* HB-1. J Chem Technol Biot.

[14] Wang D, Hwang JS, Kim DH, Lee SB, Kim DH, Joe MH (2020). A newly isolated *Bacillus siamensis* SB1001 for mass production of poly-γ-glutamic acid. Process Biochem.

[15] Wang D, Kim HM, Lee SB, Kim DH, Joe MH (2020). High-level production of poly-γ-glutamic acid from untreated molasses by *Bacillus siamensis* IR10. Microb Cell Fact.

[16] Cai D, Chen Y, He P, Wang S, Mo F, Li X, Wang Q, Nomura CT, Wen Z, Ma X, Chen S (2018). Enhanced production of poly-γ-glutamic acid by improving ATP supply in metabolically engineered *Bacillus licheniformis*. Biotechnol Bioeng.

[17] Kongklom N, Luo H, Shi Z, Pechyen C, Chisti Y, Sirisansaneeyakul S (2015). Production of poly-gamma-glutamic acid by glutamic acid-independent *Bacillus licheniformis* TISTR 1010 using different feeding strategies. Biochem Eng J.

[18] Peng Y, Jiang B, Zhang T, Mu W, Miao M, Hua Y (2015). High-level production of poly (γ-glutamic acid) by a newly isolated glutamate-independent strain *Bacillus methylotrophicus*. Process Biochem.

[19] Feng J, Gu Y, Sun Y, Han L, Yang C, Zhang W, Cao M, Song C, Gao W, Wang S (2014). Metabolic engineering of *Bacillus amyloliquefaciens* for poly-gamma-glutamic acid (γ-PGA) over production. Microb Biotechnol.

[20] Feng J, Gu Y, Quan Y, Cao M, Gao W, Zhang W, Wang S, Yang C, Song C (2015). Improved poly-γ-glutamic acid production in *Bacillus amyloliquefaciens* by modular pathway engineering. Metab Eng.

[21] Liu H, Yan Q, Wang Y, Li Y, Jiang Z (2022). Efficient production of poly-γ-glutamic acid by *Bacillus velezensis* via solid-state fermentation and its application. Food Biosci.

[22] Cai D, He P, Lu X, Zhu C, Zhu J, Zhan Y, Wang Q, Wen Z, Chen S (2017). A novel approach to improve poly-γ-glutamic acid production by NADPH regeneration in *Bacillus licheniformis* WX-02. Sci Rep.

[23] Li BC, Cai D, Chen S (2021). Metabolic engineering of central carbon metabolism of *Bacillus licheniformis* for enhanced production of poly-γ-glutamic acid. Appl Biochem Biotech.

[24] Zhu YF, Du SS, Yan YF, Pan F, Wang R, Li S, Xu H, Luo ZS (2022). Systematic engineering of *Bacillus amyloliquefaciens* for efficient production of poly-γ-glutamic acid from crude glycerol. Bioresour Technol.

[25] Zhang W, He YL, Gao WX, Feng J, Cao MF, Yang C, Song CJ, Wang SF (2015). Deletion of genes involved in glutamate metabolism to improve poly-gamma-glutamic acid production in B. amyloliquefaciens LL3. J Ind Microbiol Biotechnol..

[26] Koo BM, Kritikos G, Feralli JD, Todor H, Tong K, Kimsey H, Wapinski I, Galardini M, Cabal A, Peters JM, Hachmann AB, Rudner DZ, Allen KN, Typas A, Gross CA (2017). Construction and analysis of two genome-scale deletion libraries for *Bacillus subtilis*. Cell Syst.

[27] Jung MY, Kang MS, Lee KE, Lee EY, Park SJ (2019). *Paraburkholderia dokdonella* sp.nov., isolated from a plant from the genus Campanula. J Microbiol.

[28] Min JH, Reddy LV, Dimitris C, Kim YM, Wee YJ (2019). Optimized production of poly(γ-glutamic acid) by *Bacillus* sp. FBL-2 through response surface methodology using central composite design. J Microbiol Biotechnol.

[29] Araki R, Fujie K, Yuine N, Watabe Y, Maruo K, Suzuki H, Hashimoto K (2020). The possibility of suppression of increased postprandial blood glucose levels by gamma-polyglutamic acid-rich natto in the early phase after eating: a randomized crossover pilot study. Nutrients.

[30] Shultana R, Zuan ATK, Yusop MR, Saud HM, EI-Shehawi AM.  (2021). *Bacillus tequilensis* strain ‘UPMRB9’ improves biochemical attributes and nutrient accumulation in different rice varieties under salinity stress. PLoS ONE.

[31] Kwon HT, Lee YM, Kim JY, Balaraju K, Kim HT, Jeon YH (2022). Identification and characterization of *Bacillus tequilensis* GYUN-300: an antagonistic bacterium against red pepper anthracnose caused by *Colletotrichum acutatum* in Korea. Front Microbio.

[32] Xu M, Guo J, Li T, Zhang C, Peng X, Xing K, Qin S (2021). Antibiotic effects of volatiles produced by *Bacillus tequilensis* XK29 against the black spot disease caused by *Ceratocystis fimbriata* in postharvest sweet potato. J Agric Food Chem.

[33] Guerrero-Barajas C, Constantino-Salinas EA, Amora-Lazcano E, Tlalapango-Ángeles D, Mendoza-Figueroa JS, Cruz-Maya JA, Jan-Roblero J (2020). *Bacillus mycoides* A1 and *Bacillus tequilensis* A3 inhibit the growth of a member of the phytopathogen *Colletotrichum gloeosporioides* species complex in avocado. J Sci Food Agr.

[34] Halmschlag B, Putri SP, Fukusaki E, Blank LM (2020). Poly-γ-glutamic acid production by *Bacillus subtilis* 168 using glucose as the sole carbon source: a metabolomic analysis. J Biosci Bioeng.

[35] Jault J, Fieulaine S, Nessler S, Gonzalo P, Pietro A, Di Deutscher J, Galinier A (2000). The HPr kinase from *Bacillus subtilis* is a homo-oligomeric enzyme which exhibits strong positive cooperativity for nucleotide and fructose 1,6-bisphosphate binding. J Biol Chem.

[36] Han Y, Song J, Wang L, Shu C, Guo J, Chen L (2016). Prediction and characterization of protein-protein interaction network in *Bacillus licheniformis* WX-02. Sci Rep.

[37] Zhan YY, Sheng BJ, Wang H, Shi J, Cai DB, Yi L, Yang SH, Wen ZY, Ma X, Chen SW (2018). Rewiring glycerol metabolism for enhanced production of poly-γ-glutamic acid in *Bacillus licheniformis*. Biotechnol Biofuels.

[38] Zeng W, Chen G, Wang Q, Zheng S, Shu L, Liang Z (2014). Metabolic studies of temperature control strategy on poly (gamma-glutamic acid) production in a thermophilic strain *Bacillus subtilis* GXA-28. Bioresour Technol.

[39] Huang J, Du Y, Xu G, Zhang H, Zhu F, Huang L, Xu Z (2011). High yield and cost-effective production of poly (γ-glutamic acid) with *Bacillus subtilis*. Eng Life Sci.

[40] Gunka K, Commichau FM (2012). Control of glutamate homeostasis in *Bacillus subtilis*: a complex interplay between ammonium assimilation, glutamate biosynthesis and degradation. Mol Microbiol.

[41] Scoffone V, Dondi D, Biino G, Borghese G, Pasini D, Galizzi A, Calvio C (2013). Knockout of pgdS and ggt genes improves gamma-PGA yield in *B. subtilis*. Biotechnol Bioeng.

[42] Wang D, Kim HM, Lee SB, Kim DH, Joe MH (2020). Simultaneous production of poly-γ-glutamic acid and 2,3-butanediol by a newly isolated *Bacillus subtilis* CS13. Appl Microbiol Biot.

[43] Renna MC, Najimudin N, Winik LR, Zahler SA (1993). Regulation of the *Bacillus subtilis* alsS, alsD, and alsR genes involved in post-exponential-phase production of acetoin. J Bacteriol.

[44] Tian GM, Wang Q, Wei XT, Ma X, Chen SW (2017). Glutamate dehydrogenase (RocG) in *Bacillus licheniformis* WX-02: Enzymatic properties and specific functions in glutamic acid synthesis for poly-γ-glutamic acid production. Enzyme Microb Technol.

[45] Yang F, Liu N, Chen YZ, Wang S, Liu J, Zhao L, Ma X, Cai DB, Chen SW (2022). Rational engineering of cofactor specificity of glutamate dehydrogenase for poly-γ-glutamic acid synthesis in *Bacillus licheniformis*. Enzyme Microb Technol.

[46] Manabe K, Kageyama Y, Morimoto T, Shimizu E, Takahashi H, Kanaya S, Ara K, Ozaki K, Ogasawara N (2013). Improved production of secreted heterologous enzyme in *Bacillus subtilis* strain MGB874 via modification of glutamate metabolism and growth conditions. Microb Cell Factories.

[47] Commichau FM, Gunka K, Landmann JJ, Stülke J (2008). Glutamate metabolism in *Bacillus subtilis*: gene expression and enzyme activities evolved to avoid futile cycles and to allow rapid responses to perturbations of the system. J Bacteriol.

[48] Mahipant G, Paemanee A, Roytrakul S, Kato J, Vangnai AS (2017). The significance of proline and glutamate on butanol chaotropic stress in *Bacillus subtilis* 168. Biotechnol Biofuels.

[49] Morawska LP, Weme RGJDO, Frenzel E, Dirkzwager M, Hoffmann T, Bremer E, Kuipers OP (2022). Stress-induced activation of the proline biosynthetic pathway in *Bacillus subtilis*: a population-wide and single-cell study of the osmotically controlled *proHJ* promoter. Microb Biotechnol.

[50] Taymaz-Nikere H, Lara AR (2022). *Vitreoscilla* haemoglobin: a tool to reduce overflow metabolism. Microorganisms.

[51] Zhao C, Zhang Y, Wei X, Hu Z, Zhu F, Xu L, Luo M, Liu H (2013). Production of ultra-high molecular weight poly-gamma-glutamic acid with *Bacillus licheniformis* P-104 and characterization of its flocculation properties. Appl Biochem Biotechnol.

[52] Poo H, Park C, Kwak MS, Choi DY, Hong SP, Lee H, Lim YT, Choi YK, Bae SR, Uyama H, Kim CJ, Sung MH (2010). New biological functions and applications of high-molecular-mass poly-gamma-glutamic acid. Chem Biodivers.

[53] Yu SL, Price MA, Wang Y, Liu Y, Guo YM, Ni XM, Rosser SJ, Bi CH, Wang M (2020). CRISPR-dCas9 mediated cytosine deaminase base editing in *Bacillus subtilis*. ACS Synth Biol.

[54] Jin P, Kang Z, Yuan PH, Du GH, Chen J (2016). Production of specific-molecular-weight hyaluronan by metabolically engineered *Bacillus subtilis* 168. Metab Eng.

