# Supplementary material for: Engineering of a newly isolated Bacillus tequilensis BL01 for poly-γ-glutamic acid production from citric acid
Source: Microb Cell Fact. 2022 Dec 29;21:276. doi: 10.1186/s12934-022-01994-z (PMC9798646; doi:10.1186/s12934-022-01994-z)
Supplement: Supplementary file 1 — Additional file 1: Figure S1. Neighbor-joining tree based on the 16S rDNA gene sequence showing relationships between BL01 and Bacillus species. Bootstrap values greater than 50 are indicated at the branch nodes. Bar, 0.005 substitutions per nucleotide position. T, representative type strain. GenBank accession numbers of 16S rDNA appear in brackets. Figure S2. The γ-PGA titer normalized to the biomass of the individual cultures. (a) Effect of carbon sources; (b) Effect of nitrogen sources; (c) Effect of temperature. Values represent the mean ± SD, n=3. Figure S3. Effect of disruption of pgdS and ggt genes in the B. tequilensis BL01 strain. (a) Change curves of lactic acid with fermentation time, (b) Change curves of acetic acid with fermentation time, (c) Growth curves of 2,3-BD with fermentation time. Values represent the mean ± SD, n=3. Figure S4. Effect of disruption of sucA and alsS genes in the B. tequilensis BL01 strain. (a) Change curves of acetic acid with fermentation time, (b) Change curves of lactic acid with fermentation time. Values represent the mean ± SD, n=3. Figure S5. The glutamic acid curve of B. tequilensis BL01 and mutant strains. Values represent the mean ± SD, n=3. Figure S6. The growth curve of B. tequilensis BL01 and engineered strains in the medium without citric acid. Values represent the mean ± SD, n=3. Figure S7. Time curves of fed-batch fermentation of γ-PGA in a 5 L fermenter by BL01 and BL01ΔpgdSΔggtΔsucAΔgudB:P43-citZ-pyk strain, (a) 2,3-BD growth curves, (b) acetic acid growth curves. Data are given as the mean ± SD, n=3. Aeration rate: 10 mL/min; agitation rate: 400 rpm (0–6 h), 500 rpm (6–12 h), 600 rpm (12–24 h), 700 rpm (24–48 h); the pH was controlled at pH 6.5±0.2. 20g of sodium citrate dissolved in 50 mL sterile water was fed into the fermenter at 24h, and 150 mL of feed medium containing 700 g/L of sucrose was fed at 30 and 36 h, individually. Figure S8. The molecular weight of γ-PGA produced by (a) B. tequilensis BL01 and ( [file 12934_2022_1994_MOESM1_ESM.docx]

**Supplementary Information for**

**Engineering of a newly isolated *Bacillus tequilensis* BL01 for poly-γ-glutamic acid production from citric acid**

Dexin Wang^1,2^†, Xiaoping Fu^1,2^†, Dasen Zhou^3^, Jiaqi Gao^1,2,4^, Wenqin Bai^1,2^**^*^**

^1^ CAS Key Laboratory of Systems Microbial Biotechnology, Tianjin Institute of Industrial Biotechnology, Chinese Academy of Sciences, Tianjin, 300308, China

^2^ National Center of Technology Innovation for Synthetic Biology, Tianjin, 300308, China

^3^ College of Biotechnology, Tianjin University of Science and Technology, Tianjin, 300457, China

^4^ University of Chinese Academy of Sciences, 19A Yuquan Road, Shijingshan District, Beijing, 100049, China

**^*^**Correspondence to: WB (baiwq@tib.cas.cn)

†These authors contributed equally to this work

**Additional file**

**Figure legends**

**Fig. S1** Neighbor-joining tree based on the 16S rDNA gene sequence showing relationships between BL01 and *Bacillus* species. Bootstrap values greater than 50 are indicated at the branch nodes. Bar, 0.005 substitutions per nucleotide position. T, representative type strain. GenBank accession numbers of 16S rDNA appear in brackets.

**Fig. S2** The γ-PGA titer normalized to the biomass of the individual cultures. (a) Effect of carbon sources; (b) Effect of nitrogen sources; (c) Effect of temperature. Values represent the mean ± SD, n=3.

**Fig. S3** Effect of disruption of *pgdS* and *ggt* genes in the *B. tequilensis* BL01 strain. (a) Change curves of lactic acid with fermentation time, (b) Change curves of acetic acid with fermentation time, (c) Growth curves of 2,3-BD with fermentation time. Values represent the mean ± SD, n=3.

**Fig. S4** Effect of disruption of *sucA* and *alsS* genes in the *B. tequilensis* BL01 strain. (a) Change curves of acetic acid with fermentation time, (b) Change curves of lactic acid with fermentation time. Values represent the mean ± SD, n=3.

**Fig. S5** The glutamic acid curve of *B. tequilensis* BL01 and mutant strains. Values represent the mean ± SD, n=3.

**Fig. S6** The growth curve of *B. tequilensis* BL01 and engineered strains in the medium without citric acid. Values represent the mean ± SD, n=3.

**Fig. S7** Time curves of fed-batch fermentation of γ-PGA in a 5 L fermenter by BL01 and BL01Δ*pgdS*Δ*ggt*Δ*sucA*Δ*gudB*:*P43*-*citZ*-*pyk* strain, (a) 2,3-BD growth curves, (b) acetic acid growth curves. Data are given as the mean ± SD, n=3. Aeration rate: 10 mL/min; agitation rate: 400 rpm (0–6 h), 500 rpm (6–12 h), 600 rpm (12–24 h), 700 rpm (24–48 h); the pH was controlled at pH 6.5±0.2. 20g of sodium citrate dissolved in 50 mL sterile water was fed into the fermenter at 24h, and 150 mL of feed medium containing 700 g/L of sucrose was fed at 30 and 36 h, individually.

**Fig. S8** The molecular weight of γ-PGA produced by (a) *B. tequilensis* BL01 and (b) *B. tequilensis* BL01Δ*pgdS*Δ*ggt*Δ*sucA*Δ*gudB*:*P43*-*citZ*-*pyk* strain.


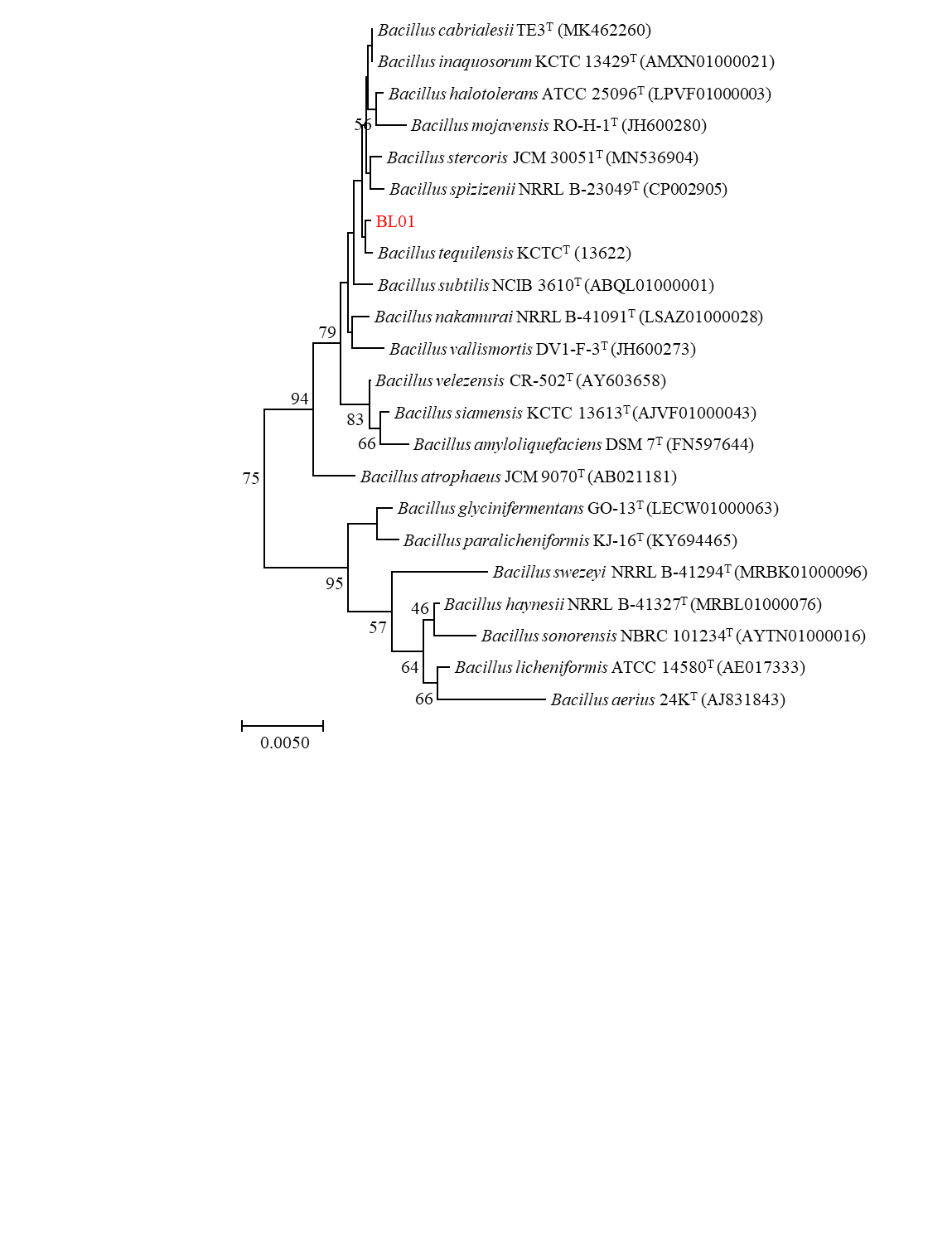


**Fig. S1** Neighbor-joining tree based on the 16S rDNA gene sequence showing relationships between BL01 and *Bacillus* species. Bootstrap values greater than 50 are indicated at the branch nodes. Bar, 0.005 substitutions per nucleotide position. T, representative type strain. GenBank accession numbers of 16S rDNA appear in brackets.

a

b

c

**Fig. S2** The γ-PGA titer normalized to the biomass of the individual cultures. (a) Effect of carbon sources; (b) Effect of nitrogen sources; (c) Effect of temperature. Values represent the mean ± SD, n=3.

b

a

c

**Fig. S3** Effect of disruption of *pgdS* and *ggt* genes in the *B. tequilensis* BL01 strain. (a) Change curves of lactic acid with fermentation time, (b) Change curves of acetic acid with fermentation time, (c) Growth curves of 2,3-BD with fermentation time. Values represent the mean ± SD, n=3.

b

a

**Fig. S4** Effect of disruption of *sucA* and *alsS* genes in the *B. tequilensis* BL01 strain. (a) Change curves of acetic acid with fermentation time, (b) Change curves of lactic acid with fermentation time. Values represent the mean ± SD, n=3.

**Fig. S5** The glutamic acid curve of *B. tequilensis* BL01 and mutant strains. Values represent the mean ± SD, n=3.

**Fig. S6** The growth curve of *B. tequilensis* BL01 and engineered strains in the medium without citric acid. Values represent the mean ± SD, n=3.

b

a

**Fig. S7** Time curves of fed-batch fermentation of γ-PGA in a 5 L fermenter by BL01 and BL01Δ*pgdS*Δ*ggt*Δ*sucA*Δ*gudB*:*P43*-*citZ*-*pyk* strain, (a) 2,3-BD growth curves, (b) acetic acid growth curves. Data are given as the mean ± SD, n=3. Aeration rate: 10 mL/min; agitation rate: 400 rpm (0–6 h), 500 rpm (6–12 h), 600 rpm (12–24 h), 700 rpm (24–48 h); the pH was controlled at pH 6.5±0.2. 20g of sodium citrate dissolved in 50 mL sterile water was fed into the fermenter at 24h, and 150 mL of feed medium containing 700 g/L of sucrose was fed at 30 and 36 h, individually.


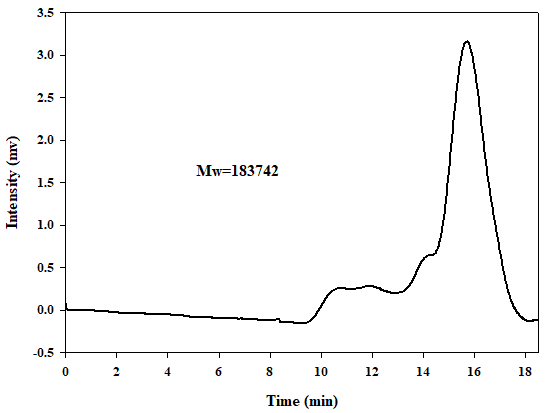


a


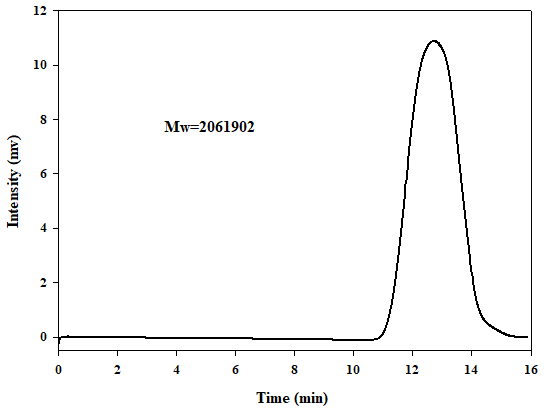


b

**Fig. S8** The molecular weight of γ-PGA produced by (a) *B. tequilensis* BL01 and (b) *B. tequilensis* BL01Δ*pgdS*Δ*ggt*Δ*sucA*Δ*gudB*:*P43*-*citZ*-*pyk* strain.

**Table S1** **Strains and plasmids used in this study**

| **Strain/plasmid** | **Genotype/description** | **Reference** |
| --- | --- | --- |
| **strains** |  |  |
| *E. coli* DH5α |  | Laboratory stock |
| *B.* *tequilensis* BL01 | Isolated from soybean | This study |
| *B.* *tequilensis* BL01Δ*pgdS* | *B.* *tequilensis* BL01 derivate, deletion of *pgdS* | This study |
| *B.* *tequilensis* BL01Δ*ggt* | *B.* *tequilensis* BL01 derivate, deletion of *ggt* | This study |
| *B.* *tequilensis* BL01Δ*pgdS* Δ*ggt* | *B.* *tequilensis* BL01 derivate, deletion of *pgdS* and *ggt* | This study |
| *B.* *tequilensis* BL01Δ*alsS* | *B.* *tequilensis* BL01 derivate, deletion of *alsS* | This study |
| *B.* *tequilensis* BL01Δ*sucA* | *B.* *tequilensis* BL01 derivate, deletion of *sucA* | This study |
| *B.* *tequilensis* BL01Δ*gudB* | *B.* *tequilensis* BL01 derivate, deletion of *gudB* | This study |
| *B.* *tequilensis* BL01Δ*rocG* | *B.* *tequilensis* BL01 derivate, deletion of *rocG* | This study |
| *B.* *tequilensis* BL01Δ*proB* | *B.* *tequilensis* BL01 derivate, deletion of *proB* | This study |
| *B.* *tequilensis* BL01Δ*gudB* Δ*rocG* | *B.* *tequilensis* BL01 derivate, deletion of *gudB* and *rocG* | This study |
| *B.* *tequilensis* BL01Δ*pgdS* Δ*sucA* | *B.* *tequilensis* BL01 derivate, deletion of *pgdS* and *sucA* | This study |
| *B.* *tequilensis* BL01Δ*pgdS* Δ*ggt*Δ*gudB* | *B.* *tequilensis* BL01 derivate, deletion of *pgdS*, *ggt*, and *gudB* | This study |
| *B.* *tequilensis* BL01Δ*pgdS* Δ*ggt*Δ*sucA* | *B.* *tequilensis* BL01 derivate, deletion of *pgdS*, *ggt*, and *sucA* | This study |
| *B.* *tequilensis* BL01Δ*pgdS* Δ*ggt*Δ*sucA*Δ*gudB* | *B.* *tequilensis* BL01 derivate, deletion of *pgdS*, *ggt*, *sucA* and *gudB* | This study |
| *B.* *tequilensis* BL01:*P43*-*citZ* | *B.* *tequilensis* BL01 derivate, carrying *P43*-*citZ* | This study |
| *B.* *tequilensis* BL01:*P43*-*icd* | *B.* *tequilensis* BL01 derivate, carrying *P43*-*icd* | This study |
| *B.* *tequilensis* BL01:*P43*-*gltA* | *B.* *tequilensis* BL01 derivate, carrying *P43*-*gltA* | This study |
| *B.* *tequilensis* BL01:*P43*-*pdhABCD* | *B.* *tequilensis* BL01 derivate, carrying *P43*-*pdhABCD* | This study |
| *B.* *tequilensis* BL01:P43-pycA | *B.* *tequilensis* BL01 derivate, carrying *P43*-*pycA* | This study |
| *B.* *tequilensis* BL01:*P43*-*pyk* | *B.* *tequilensis* BL01 derivate, carrying *P43*-*pyk* | This study |
| *B.* *tequilensis* BL01:*P43*-*pgsBCAE* | *B.* *tequilensis* BL01 derivate, carrying *P43*-*pgsBCAE* | This study |
| *B.* *tequilensis* BL01:*P43*-*citZ*-*icd*-*gltA* | *B.* *tequilensis* BL01 derivate, carrying *P43*-*citZ*-*icd*-gltA | This study |
| *B.* *tequilensis* BL01:*P43*-*citZ*-*pyk* | *B.* *tequilensis* BL01 derivate, carrying *P43*-*citZ*-*pyk* | This study |
| *B.* *tequilensis* BL01ΔpgdS Δ*ggt*Δ*sucA*Δ*gudB*:*P43*-*pdhABCD* | *B.* *tequilensis* BL01Δ*pgdS*Δ*ggt*Δ*sucA*Δ*gudB* derivate, carrying *P43*-*pdhABCD* | This study |
| *B.* *tequilensis* BL01Δ*pgdS* Δ*ggt*Δ*sucA*Δ*gudB*:*P43*-*pgsBCAE* | *B.* *tequilensis* BL01Δ*pgdS*Δ*ggt*Δ*sucA*Δ*gudB* derivate, carrying *P43*-*pgsBCAE* | This study |
| *B.* *tequilensis* BL01Δ*pgdS* Δ*ggt*Δ*sucA*Δ*gudB*:*P43*-citZ-*pyk* | *B.* *tequilensis* BL01Δ*pgdS*Δ*ggt*Δ*sucA*Δ*gudB* derivate, carrying *P43*-*citZ*-*pyk* | This study |
| **Plasmids** |  |  |
| pBAC-cas9 | *E. coli*-*B. subtilis* shuttle vector, Amp^r^ (*E. coli*), Cm^r^ (*B. subtilis*), containing fusion proteins of Cas9 | [53] |
| pBAC-cas9-N20-*pgdS*-up-down | pBAC-cas9 derivate, containing *pgdS* targeting 20 bp gRNA and 2 kb donor DNA | This study |
| pBAC-cas9-N20-*ggt*-up-down | pBAC-cas9 derivate, containing *ggt* targeting 20 bp gRNA and 2 kb donor DNA | This study |
| pBAC-cas9-N20-*alsS*-up-down | pBAC-cas9 derivate, containing *alsS* targeting 20 bp gRNA and 2 kb donor DNA | This study |
| pBAC-cas9-N20-*sucA*-up-down | pBAC-cas9 derivate, containing *sucA* targeting 20 bp gRNA and 2 kb donor DNA | This study |
| pBAC-cas9-N20-*gudB*-up-down | pBAC-cas9 derivate, containing *gudB* targeting 20 bp gRNA and 2 kb donor DNA | This study |
| pBAC-cas9-N20-*rocG*-up-down | pBAC-cas9 derivate, containing *rocG* targeting 20 bp gRNA and 2 kb donor DNA | This study |
| pBAC-cas9-N20-*proB*-up-down | pBAC-cas9 derivate, containing *proB* targeting 20 bp gRNA and 2 kb donor DNA | This study |
| *pP43NMK* | Amp^r^, Km^r^, *E. coli*-*B. subtilis* shuttle vector, P43-MCS | [54] |
| *P43*-*gltA* | pP43NMK derivate, containing the gene *gltA* | This study |
| *P43*-icd | pP43NMK derivate, containing the gene *icd* | This study |
| *P43*-*citZ* | pP43NMK derivate, containing the gene *citZ* | This study |
| *P43*-*pdhABCD* | pP43NMK derivate, containing the gene *pdhABCD* | This study |
| *P43*-*pycA* | *pP43NMK* derivate, containing the gene *pycA* | This study |
| *P43*-*pyk* | *pP43NMK* derivate, containing the gene *pyk* | This study |
| *P43*-*pgsBCAE* | *pP43NMK* derivate, containing the gene *pgsBCAE* | This study |
| *P43*-*citZ*-*pyk* | *pP43NMK* derivate, containing the gene *citZ* and *pyk* | This study |
| *P43*-*citZ*-*icd*-*gltA* | *pP43NMK* derivate, containing the gene *citZ*, *icd* and *gltA* | This study |

**Table S2 Primers used in this study**

| **Primer** | **Sequence (5’-3’)** |
| --- | --- |
| *pgdS*-N20-F | ATGTAAAGAGGGTTTCGATCCATC |
| *pgdS*-N20-R | AAACGATGGATCGAAACCCTCTTT |
| *pgdS*-up-F | AATCTGGCCTTATTGGCCAAAGATACGCTTGGAGAATTTGC |
| *pgdS*-up-R | CTTTATGTTCTTTTATTATCTCCTCCTCTTTTTGC |
| *pgdS*-down-F | GATAATAAAAGAACATAAAGCCTGGTGATGTTG |
| *pgdS*-down-R | GAGATGGCCAACGAGGCCCCTCATATCGAATCGGTTTTTGAC |
| *ggt*-N20-F | ATGTACTaCTCCTCCTCCAAGCTC |
| *ggt*-N20-R | AAACGAGCTTGGAGGAGGAGtAGT |
| *ggt*-up-F | AATCTGGCCTTATTGGCCGAAATCTGACGCTCCCAATTTTC |
| *ggt*-up-R | CTGTAAAGTGTAATTAATTGCTTCATCGAACGGG |
| *ggt*-down-F | CAATTAATTACACTTTACAGTTGCTGATCGATG |
| *ggt*-down-R | GAGATGGCCAACGAGGCCTCGTGACAGGGTCAAAAGATTAC |
| *alsS*-N20-F | ATGTGTTTCTGTCTCTGGTGACGG |
| *alsS*-N20-R | AAACCCGTCACCAGAGACAGAAAC |
| *alsS*-up-F | AATCTGGCCTTATTGGCCATCGTTTAAAACGGACACATCAAT |
| *alsS*-up-R | AACAGAACGGTTTCTCCCGATTTCACCAATGAAG |
| *alsS*-down-F | TCGGGAGAAACCGTTCTGTTCATTTACGTTCTTT |
| *alsS*-down-R | GAGATGGCCAACGAGGCCCCTGAAATGGTTTCAATAGCTCAC |
| *sucA*-N20-F | ATGTGCTCTTTGAAGTCATGCACA |
| *sucA*-N20-R | AAACTGTGCATGACTTCAAAGAGC |
| *sucA*-up-F | AATCTGGCCTTATTGGCCTCACTGTCATGAAGCACAAGGTTT |
| *sucA*-up-R | CTGATTCAAATTCAAGACGCTGAAACAAAATCAGC |
| *sucA*-down-F | GCGTCTTGAATTTGAATCAGATCGGCTGTAACAC |
| *sucA*-down-R | GAGATGGCCAACGAGGCCTTCGACAAAATGGACGAAACAAAAC |
| *gudB*-N20-F | ATGTAAACGTATCCCGCCTTTCGT |
| *gudB*-N20-R | AAACACGAAAGGCGGGATACGTTT |
| *gudB*-up-F | AATCTGGCCTTATTGGCCGCGGCATATCTGATCAGCAACG |
| *gudB*-up-R | TGCCAAATAGTTGAGTTAACCTCCTAGAATCTTCTG |
| *gudB*-down-F | GTTAACTCAACTATTTGGCAAAATTTATGCATGAT |
| *gudB*-down-R | GAGATGGCCAACGAGGCCGCGTTTTCGTCACTTTATGCAC |
| *proB*-N20-F | ATGTACCTCGGGTGCCGTAGCGGC |
| *proB*-N20-R | AAACGCCGCTACGGCACCCGAGGT |
| *proB*-up-F | AATCTGGCCTTATTGGCCCCTCTGTAGCGGATAAATTCGAC |
| *proB*-up-R | TTTTTGCTCTTTATTCTCCTCCGCGGCTCTTCTA |
| *proB*-down-F | AGGAGAATAAAGAGCAAAAACTTGCGGACATT |
| *proB*-down-R | GAGATGGCCAACGAGGCCACGTCTAAACCATCATTTAACGTAA |
| *rocG*-N20-F | ATGTAAGCATCGCGTTTCCGCGGA |
| *rocG*-N20-R | AAACTCCGCGGAAACGCGATGCTT |
| *rocG*-up-F | AATCTGGCCTTATTGGCCAGCGGTAGAATTTCAATTTCCTG |
| *rocG*-up-R | GCTTCTCAAAAATAAAGCCCGGAGAATCGAATTC |
| *rocG*-down-F | GGGCTTTATTTTTGAGAAGCCTCCGCAAAATC |
| *rocG*-down-R | GAGATGGCCAACGAGGCCACCTGTAAATGAGACAAACCGTG |
| *citZ*(P43)-F | GAATGTACACATGACAGCGACACGCGGTCT |
| *citZ*(P43)-R | GCTTTCATCATTAGGCTCTTTCTTCAATCGGAACG |
| P43(*citZ*)-back-F | AAGAGCCTAATGATGAAAGCTTGGCGTAATCAT |
| P43(*citZ*)-back-R | TCGCTGTCATGTGTACATTCCTCTCTTACCTATAATG |
| *icd*(P43)-F | CGGGATCCATGGCACAAGGTGAAAAAATTACAG |
| *icd*(P43)-R | TCCCCGCGGTTAGTCCATGTTTTTGATCAGTTCTTC |
| P43(*icd*)-F | GGTCTCACCGCGGTGAAAGCTTGGCGTAATCATG |
| P43(icd)-R | GGTCTCGGATCCGATTTCCTCCTCTATAATGGTACCGCTATCAC |
| *gltA*(P43)-F | GAATGTACACATGACGTACAATCAAATGCCAAAAG |
| *gltA*(P43)-R | GCTTTCATCATTACTGTACTACCGCTTGTTTTTGT |
| P43(*gltA*)-back-F | AGTACAGTAATGATGAAAGCTTGGCGTAATCAT |
| P43(*gltA*)-back-R | TGTACGTCATGTGTACATTCCTCTCTTACCTATAATG |
| *pgsBCAE*(P43)-F | GAATGTACACATGTGGTTACTCATTATAGCCTGT |
| *pgsBCAE*(P43)-R | CAAGCTTTCATTATTTATTGGCGCTTACCGGTTC |
| P43(*pgsBCAE*)-F | CAATAAATAATGAAAGCTTGGCGTAATCATGGTC |
| P43(*pgsBCAE*)-R | GTAACCACATGTGTACATTCCTCTCTTACCTATAATG |
| *pdhABCD*(P43)-F | GAATGTACACATGGCTGCAAAAACGAAAAAAG |
| *pdhABCD*(P43)-R | GCTTTCATCATTATTTTACGATGTGAATCGGACTTC |
| P43(*pdhABCD*)-F | CGTAAAATAATGATGAAAGCTTGGCGTAATCAT |
| P43(*pdhABCD*)-R | TTGCAGCCATGTGTACATTCCTCTCTTACCTATAATG |
| *pycA*(P43)-F | GAATGTACACTTGTCTCAGCAATCGATACAAAAAG |
| *pycA*(P43)-R | GCTTTCATCATTATGCTTTTTCAATTTCGAGAAGC |
| P43(*pycA*)-back-F | AAAAGCATAATGATGAAAGCTTGGCGTAATCAT |
| P43(*pycA*)-back-R | GCTGAGACAAGTGTACATTCCTCTCTTACCTATAATG |
| *pyk*(P43)-F | GAATGTACACATGAGAAAAACTAAAATTGTTTGTACCATC |
| *pyk*(P43)-R | GCTTTCATCATTAAAGAACGCTCGCACGGC |
| P43(*pyk*)-back-F | CGTTCTTTAATGATGAAAGCTTGGCGTAATCAT |
| P43(*pyk*)-back-R | TTTTTCTCATGTGTACATTCCTCTCTTACCTATAATG |
| *citZ*(P43-*pyk*)-F | GCCGTGCGAGCGTTCTTTAAGTAAGAGAGGAATGTACACATGACAGCGACACGCGGTCT |
| *pyk*(P43-*citZ*)-R | TTAAAGAACGCTCGCACGGC |
| *icd*-F | GTAAGAGAGGAATACTAGTATGGCACAAGGTGAAAAAATTACAG |
| *CitZ*-R | TACTAGTATTCCTCTCTTACTTAGGCTCTTTCTTCAATCGGAAC |
| *gltA*-F | TCAAAAACATGGACTAAGTAAGAGAGGAATGTACACATGACGTACAATCAAATGCCAA |
| *icd*-R | TACTTAGTCCATGTTTTTGATCAGTTCTTC |
| *gltA*-R | ATCATCTAGATTACTGTACTACCGCTTGTTTTTGT |
| P43(*citZ*-*icd*-*gltA*) -F | AGTACAGTAATCTAGATGATGAAAGCTTGGCGTAATC |

**Table S3 γ-PGA production by the isolated strains**

| **Strains** | **24 h** | | **γ-PGA/Biomass**  **(g/g)** | **species** |
| --- | --- | --- | --- | --- |
|  | **Biomass (g/L)** | **γ-PGA (g/L)** |  |  |
| BL01 | 4.5 ± 0.2 | 8.6 ± 0.3 | 1.91 ± 0.16 | *B. tequilensis* |
| F29 | 4.7 ± 0.3 | 8.3± 0.3 | 1.76 ± 0.19 | *B. halotolerans* |
| L29 | 5.1 ± 0.3 | 7.9 ± 0.3 | 1.55 ± 0.16 | *B. zanthoxyli* |
| L45 | 4.8 ± 0.2 | 7.4 ± 0.3 | 1.54 ± 0.13 | *B. licheniformis* |
| W1 | 6.1 ± 0.3 | 5.1 ± 0.2 | 0.84 ± 0.07 | *B. subtilis* |

Note: The biomass, γ-PGA titer and γ-PGA titer/Biomass were measured at 24 h. Values represent the mean ± SD, n=3.
